# Mechanisms and Consequences of Dopamine Depletion-Induced Attenuation of the Spinophilin/Neurofilament Medium Interaction

**DOI:** 10.1155/2017/4153076

**Published:** 2017-05-28

**Authors:** Andrew C. Hiday, Michael C. Edler, Asma B. Salek, Cameron W. Morris, Morrent Thang, Tyler J. Rentz, Kristie L. Rose, Lisa M. Jones, Anthony J. Baucum

**Affiliations:** ^1^Department of Biology, Indiana University-Purdue University Indianapolis, 723 W. Michigan St., Indianapolis, IN 46202, USA; ^2^Department of Chemistry and Chemical Biology, Indiana University-Purdue University Indianapolis, 723 W. Michigan St., Indianapolis, IN 46202, USA; ^3^Neuroscience Undergraduate Program, Indiana University-Purdue University Indianapolis, 723 W. Michigan St., Indianapolis, IN 46202, USA; ^4^Department of Psychology, Indiana University-Purdue University Indianapolis, 723 W. Michigan St., Indianapolis, IN 46202, USA; ^5^Molecular Physiology and Biophysics, Vanderbilt University School of Medicine University, 724 Robinson Research Building, 23rd Ave South at Pierce, Nashville, TN 37232, USA; ^6^Department of Biochemistry and the Mass Spectrometry Research Center, Vanderbilt University School of Medicine University, 465 21st Ave S. Room 9160, MRB III, Nashville, TN 37232, USA; ^7^Stark Neurosciences Research Institute, Indiana University School of Medicine, Indiana, IN, USA

## Abstract

Signaling changes that occur in the striatum following the loss of dopamine neurons in the Parkinson disease (PD) are poorly understood. While increases in the activity of kinases and decreases in the activity of phosphatases have been observed, the specific consequences of these changes are less well understood. Phosphatases, such as protein phosphatase 1 (PP1), are highly promiscuous and obtain substrate selectivity via targeting proteins. Spinophilin is the major PP1-targeting protein enriched in the postsynaptic density of striatal dendritic spines. Spinophilin association with PP1 is increased concurrent with decreases in PP1 activity in an animal model of PD. Using proteomic-based approaches, we observed dopamine depletion-induced decreases in spinophilin binding to multiple protein classes in the striatum. Specifically, there was a decrease in the association of spinophilin with neurofilament medium (NF-M) in dopamine-depleted striatum. Using a heterologous cell line, we determined that spinophilin binding to NF-M required overexpression of the catalytic subunit of protein kinase A and was decreased by cyclin-dependent protein kinase 5. Functionally, we demonstrate that spinophilin can decrease NF-M phosphorylation. Our data determine mechanisms that regulate, and putative consequences of, pathological changes in the association of spinophilin with NF-M that are observed in animal models of PD.

## 1. Introduction

Proper neuronal communication is essential for normal brain function. In animal models of Parkinson disease (PD) and in human PD patients, there is a loss of dopamine (DA) neurons that project from the substantia nigra to the striatum [1, 2]. These dopamine neurons form synapses with dendritic spines on striatal medium spiny neurons (MSNs). Loss of DA signaling leads to decreases in the number of spines on striatal MSNs [3–5] as well as losses in measures of synaptic plasticity, specifically long-term depression (LTD) [1, 6]. Changes in the organization and function of key synaptic proteins that reside in striatal MSN spines are critical in regulating changes in spine shape and number as well as LTD [7, 8].

Alterations in kinase and phosphatase activity are observed in animal models of PD [8–10]. Specifically, DA depletion decreases the activity of protein phosphatase 1 (PP1) in striatal MSNs [8]. Multiple targeting/scaffolding proteins regulate PP1 activity [11–13]. Spinophilin, the most abundant PP1-associated protein in the PSD [14], targets PP1 to functionally regulate glutamate receptors [15, 16]. Decreased activity of PP1 in animal models of PD occurs concurrently with increased association between spinophilin and PP1 [8]. Spinophilin is known to modulate synaptic plasticity as spinophilin knockout mice do not undergo corticostriatal LTD [17]. Moreover, spinophilin has been implicated as a major hub that interacts with multiple proteins that are known to be disrupted in neurodegenerative diseases [18]; however, it is not known if spinophilin binding to proteins in addition to PP1 is altered in the striatum in an animal model of PD.

We have previously found that multiple intermediate neurofilament proteins specifically coimmunoprecipitate with spinophilin [19]. Recent studies demonstrate that neurofilament proteins are found in dendritic spines [20]. Neurofilament medium (NF-M) has been shown to interact with the DA D1 receptor (D1R) to regulate D1R-mediated behaviors [20]. Moreover, NF-M is hypophosphorylated in dendritic spines compared to NF-M in the dendritic shaft, suggesting that NF-M hyposphosphorylation may be important in maintaining NF-M in the dendritic spine [20]. Both protein phosphatases 2A and PP1 have been shown to associate with and dephosphorylate neurofilament proteins [21]. However, the roles of scaffolding proteins such as spinophilin in regulating NF-M phosphorylation have never been evaluated.

Here, we have utilized an unbiased, proteomic-based approach to identify alterations in the spinophilin interactome in the striatum of an animal model of PD and demonstrate that perturbations in the spinophilin/NF-M interaction may be due to alterations in kinase activity during DA depletion. Moreover, we have found that spinophilin can regulate NF-M phosphorylation in a heterologous cell system, and therefore, we predict that spinophilin may be important in maintaining NF-M in a hypophosphorylated state in vivo.

## 2. Materials and Methods

### 2.1. Generating DNA Constructs

The templates for the constructs used were as follows: human spinophilin (REFSEQ: NM_032595.4)—modified from Dr. Maria Vivo (University of Naples “Federico II”); human NF-M (Uniprot ID P07197; Transomics BC096757-seq); human PKAc—pDONR223-PRKACA; human CDK5—pDONR223-CDK5; and human p35—pDONR223-CDK5SR1 were kind gifts from William Hahn & David Root [22] (plasmid numbers 23495, 23699, and 23779, Addgene, Cambridge, MA). PCR products were generated from these template DNAs and inserted into pDONR221 (ThermoFisher, Waltham MA) for Gateway cloning into either pcDNA3.1/nV5-DEST (NF-M) or modified pcDNA3.1/nV5-DEST vectors containing an HA-tag (spinophilin), myc-tag (PKAc or p35), or FLAG-tag (CDK5) in place of the V5 tag. Mutant spinophilin (Ser17Ala) or PKA (Lys72His) constructs were generated using QuickChange mutagenesis (Agilent Technologies, Santa Clara, CA). PCR was performed with Q5 Hot Start TAQ (New England Biolabs, Ipswich, MA) or VAPRase (Vanderbilt Antibody Protein Resource, Vanderbilt University, Nashville, TN). All constructs and mutations were sequence validated (Genewiz, South Plainfield, NJ).

### 2.2. Mammalian Cell Protein Expression

Human embryonic kidney 293FT cells (HEK293; ThermoFisher) were transfected in 25 cm^2^ flat bottom flasks with each relevant DNA construct in 500 *μ*L serum-free Dulbecco's modified Eagle's medium (DMEM) using PolyJet reagent (SignaGen Laboratories Rockville, MD) in a 3 : 1 (reagent volume : DNA mass) ratio. Empty vector DNAs were used to transfect equal DNA concentrations. Cells were incubated overnight, medium was aspirated from each flask, and cells were washed with cold phosphate-buffered saline (PBS). HEK293 cells were then suspended in a low ionic Tris homogenization buffer (0.01 M DTT, 0.005 M EDTA, 0.002 M Tris-HCl pH 7.5, 1% Triton X-100) containing protease inhibitors (HALT; ThermoFisher) and phosphatase inhibitors (20 mM sodium fluoride, 20 mM sodium orthovanadate, 20 mM *β*-glycerophosphate, and 10 mM sodium pyrophosphate; Sigma-Aldrich, St. Louis, MO or ThermoFisher).

### 2.3. Unilateral 6-Hydroxydopamine Lesions

All animal studies were carried out in accordance with the Guide for the Care and Use of Laboratory Animals and under the oversight of either the Vanderbilt University Medical Center or the Indiana University-Purdue University Indianapolis Institutional Animal Care and Use Committee. Male C57Bl/6 mice (Jackson Laboratories, Bar Harbor, ME; ~3 months of age) were lesioned with 6-hydroxydopamine. Animals were injected (i.p.) with 7.5 mg/kg of desmethylimipramine (DMI) 15 minutes prior to surgeries. Animals were then placed in an anesthesia chamber, and isoflourane was administered at a 3% concentration with a flow rate of ~1 L/minute. Animals were immobilized in ear bars attached to a stereotaxic instrument (Leica Biosystems, Buffalo Grove, IL). The top of the head was shaved, and the incision site was wiped with alcohol then iodine three times. A second injection of DMI was then given. A 1 cm incision was made, and the skin was pulled back, exposing the skull. Using a sterilized drill tip, the skull was drilled at the appropriate coordinates from Bregma (posterior—2.22 mm, lateral—1.66 mm) to target the substantia nigra. The syringe was lowered to the appropriate depth (ventral—4.7 mm). For lesion surgeries, a total volume of 800 nL of 6-hydoxydopamine hydrogen bromide (Sigma-Aldrich) at a concentration of 6.0 *μ*g/*μ*L (4.0 *μ*g/*μ*L free base) in 0.02% ascorbic acid (Sigma-Aldrich) in saline was injected at a rate of 40 nL/minute. Following injection and removal of the needle, the incision was sutured closed. To ensure animal health, 1 mL of warm saline and 5 mg/kg (animal weight × 5 *μ*L of 1 mg/mL solution) ketoprofen were injected subcutaneously. Animals were allowed to recover on a warm heating pad. Mice were sacrificed three weeks following lesion and both lesioned (ipsilateral; experimental) and intact (contralateral; control) striata were harvested.

### 2.4. Subcellular Fractionation

Lesioned or intact striatum was homogenized in a KCl buffer (without EDTA) containing protease and phosphatase inhibitors [23]. An increasing stringency of detergents was used to fractionate striatal lysates into a cytosolic, membrane-associated, and synaptic fraction as previously described [23–25]. Synaptic and extrasynaptic fractions were utilized for spinophilin immunoprecipitations (see below). The cytosolic and membrane fractions were immunoblotted for TH, and only those animals with a > 90% reduction in TH immunoreactivity (indicating efficient lesioning) were used in these studies.

### 2.5. Immunoprecipitation Assays

HEK293 cell lysate (400–600 *μ*L), whole forebrains, or dissected striatum from adult male or female wild type C57Bl/6 mice (2.5–9 months old) were homogenized and sonicated. HEK293 cell lysates were lysed in a low-ionic Tris buffer (0.01 M DTT, 0.005 M EDTA, 0.002 M Tris-HCl pH 7.5, 1% Triton X-100), whereas brain lysates were generated in a KCl/EDTA buffer (150 mM KCl, 0.01 M DTT, 0.005 M EDTA, 0.05 M Tris-HCl pH 7.5, 1% Triton X-100). Both buffers contained protease inhibitors (HALT; ThermoFisher Scientific) and phosphatase inhibitors (20 mM sodium fluoride, 20 mM sodium orthovanadate, 20 mM *β*-glycerophosphate, and 10 mM sodium pyrophosphate; Sigma-Aldrich or ThermoFisher). Lysates were added to a microcentrifuge tube with the appropriate primary antibody (goat spinophilin antibody (SC-14774, Santa Cruz Biotechnology, Dallas Texas), NF-M antibody (#2383, Cell Signaling), V5 antibody (#A190-119A Bethyl Laboratories, Montgomery, TX), HA antibody (#A190-107A Bethyl Laboratories), PP1*γ* antibody (SC-6108, Santa Cruz Biotechnology), or goat IgG (#005-000-003 Jackson Immunologicals, West Grove, PA)) and incubated at 4°C for 1 h to overnight. Protein G magnetic beads were then added to the samples and incubated 2 h to overnight at 4°C with rotation. Beads were then washed three times with immunoprecipitation (IP) buffer (150 mM NaCl, 50 mM Tris-HCl pH 7.5, 0.5% (*v*/*v*) Triton X-100). Washed beads were incubated with 2× Laemmli sample buffer, heated at 70°C for 10 minutes, separated by SDS-PAGE, and immunoblotted.

### 2.6. Immunoblotting

Immunoblotting was performed as previously described [26]. Briefly, inputs or immunoprecipitates were separated by SDS-PAGE and blotted using antibodies to spinophilin, NF-M, V5-tag, HA-tag (as above or rabbit HA (SC-805, Santa Cruz Biotechnology)), PP1*γ*1, pan PP1 (SC-7482, Santa Cruz Biotechnology), PKA substrate antibody (#9624, Cell Signaling), Myc (sc-40, Santa Cruz Biotechnology), or tyrosine hydroxylase (TH) antibody (#22941, ImmunoStar, Hudson, WI). Appropriate infrared secondary antibodies were used (donkey anti-goat, donkey anti-rabbit, or donkey anti-mouse conjugated to Alexa Fluor 690 or 780; ThermoFisher or Jackson Immunologicals), and fluorescence intensity measurements were made using Image Studio (LI-COR Biosciences, Lincoln, NE).

### 2.7. Mass Spectrometric Analysis

Spinophilin or NF-M immunoprecipitates were resolved by SDS-PAGE and stained with colloidal blue or Imperial Stain (ThermoFisher). Multiple gel regions were excised and processed. Gel segments were processed using either collisional-induced dissociation on an Orbitrap Velos mass spectrometer (ThermoFisher; Vanderbilt University Mass Spectrometry Research Center; Table S1 available online at https://doi.org/10.1155/2017/4153076) as previously described [23] or using higher-energy collisional dissociation (HCD) on a Q-Exactive mass spectrometer (ThermoFisher; Dr. Lisa Jones, Department of Chemistry, IUPUI). Conditions for MS analysis on the Q-Exactive were as follows. Digested samples were loaded onto a 100 *μ*m × 2 cm Acclaim PepMap100 C18 nanotrap column (5 *μ*m, 100 Å) (ThermoFisher) with an Ultimate 3000 liquid chromatograph (ThermoFisher) at 5 *μ*L/min. The peptides were separated on a silica capillary column that was custom-packed with C18 reverse phase material (Magic, 0.075 mm × 150 mm, 5 *μ*m, 120 Å, Michrom Bioresources Inc. Auburn, CA). The gradient was pumped at 300 nL/min from 10 to 45% solvent B (99.9% acetonitrile, 0.1% formic acid) for 87 min and then to 90% solvent B for 5 min and re-equilibrated to solvent A (99.9% water, 0.1% formic acid) for 12 min. The mass spectrometry was performed on a Q-Exactive Orbitrap. The mass spectrometer was operated in data-dependent acquisition mode controlled by the Xcalibur 2.2 software. Peptide mass spectra were acquired from an *m*/*z* range of 350–2000 at resolving power of 70,000 for 400 *m/z* ions. The top 15 most abundant multiply charged ions were subjected to higher-energy collisional dissociation (HCD) at a resolving power of 17,500 for 400 *m/z* ions. Ions with a charge state >+6 were rejected. AGC targets were set to 3e6 for MS1 and 1e5 for data-dependent MS2 with an underfill ratio of 2.5%, given an intensity threshold of 5.0e4. A dynamic exclusion of 10.0 s was used.

### 2.8. Extracted Ion Chromatogram (XIC) Analysis

CID MS/MS matching spinophilin peptides containing Ser17 and Ser100 were validated and hand annotated for b- and y-series ions. Additional spinophilin phosphopeptides were not consistently detected across all biological replicates. Accurate mass measurements were used to generate extracted ion chromatograms (XICs) with a 10-part per million tolerance. Monoisotopic *m/z* values of observed precursor ions (across different charge states) were used to generate XICs. The abundance of each phosphorylated and nonphosphorylated peptide pair was calculated as previously described [23], and a ratio was calculated by dividing the area under the curve (AUC) of the XIC of the phosphorylated sample by the sum of the AUC of the XIC of the phosphorylated plus nonphosphorylated sample (phospho/phospho + total). A ratio of the intact/lesion sample was then calculated by dividing the individual lesion value by its corresponding intact value.

CID MS/MS spectra matching NF-M or spinophilin were hand annotated. The AUCs of XICs for precursor ions of two specific tryptic peptides matching NF-M (QASHAQLGDAYDQEIR and VQSLQDEVAFLR) were used to compare the abundance of NF-M in the spinophilin immunoprecipitates isolated from intact and lesioned striatal synaptic fractions. AUCs of the XICs for the NF-M peptides were normalized to an average of the AUC of the XIC matching 3 spinophilin peptides (AAGAPQVNSK, VLEESELAR, and SVPAASGGDKEAVAR). A ratio of the intact/lesion sample was then calculated by dividing the individual lesion value by its corresponding intact value.

HCD MS/MS spectra of NF-M phosphopeptides containing Ser30, Ser346, Ser615/620, Ser628/633/641/646/654/659, Ser680/685, Ser736, and Ser837, were manually validated, and the AUCs of XICs matching these peptides were generated (Figure S1A-S1G). These AUCs were normalized either to the nonphosphorylated peptide or, if the nonphosphorylated peptide was not detected, to the nonphosphorylated NF-M peptide EQLQGLNDR. Given the repeat of certain portions of NF-M, it is not possible to delineate phosphorylation at specific sites. Phosphorylation sites on the detected tryptic peptides are shown in Figure S2.

### 2.9. Data and Statistical Analyses

A 1-column *t*-test compared to a theoretical value of 1 was used for statistical analyses comparing two normalized groups. To determine the effect of CDK5 on wildtype and S17A mutant spinophilin, the fluorescence intensity for NF-M in the HA (spinophilin) immunoprecipitate was normalized to fluorescence intensity of spinophilin in the HA IP. To account for any differences in NF-M expression, this value was subsequently divided by the fluorescence intensity for NF-M in the corresponding input. This is the fluorescence intensity formula: (coprecipitated protein/precipitated protein)/(coprecipitated protein in the input). To compare across multiple gels, a ratio of these normalized values was created by dividing each value by the wild type, no p35 condition. A two-way ANOVA followed by an uncorrected Fisher's least significant difference post hoc test was used to compare the effect of p35 and S17A genotype on the spinophilin/NF-M interaction. For all tests, a value of *p* ≤ 0.05 was considered statistically significant. Statistical analyses and graphing were performed using Prism (GraphPad, LaJolla, CA). All data are plotted as the mean ± the standard error of the mean. Grubbs outlier tests were performed on MS data comparing NF-M phosphorylation in the absence and presence of spinophilin. One outlier was removed from AKsPVPKsPVEEAK, GKsPVPKsPVEEK, and AESPVKEEAVAEVVTITK peptides.

## 3. Results

### 3.1. Proteomic Identification of Spinophilin Interacting Proteins Isolated from Extrasynaptic and Synaptic Fractions of the Striatum of 6-Hydroxydopamine Nigral Lesioned Mice

**Figure 1:**
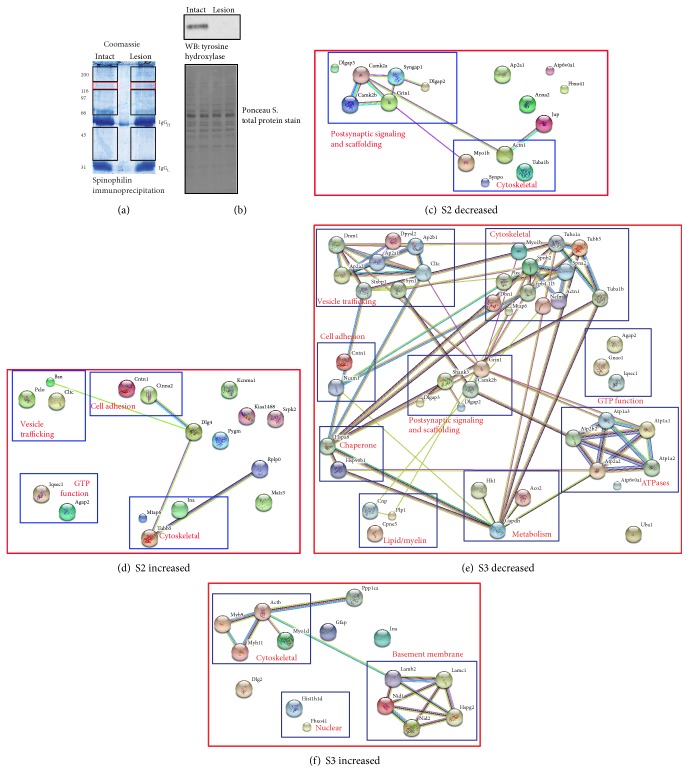
STRING map of proteins with altered expression in spinophilin immunoprecipitates isolated from DA lesioned compared to intact striatal lysates. The substantia nigra of adult C57Bl6 mice was unilaterally lesioned with 6-OHDA. Striata from the intact and lesioned hemispheres were collected, fractionated into a membrane and synaptic fraction, and immunoprecipitated for spinophilin using a goat spinophilin antibody. (a) The regions of the gel containing spinophilin and NF-M (red boxes) and spinophilin interacting proteins (black boxes) were excised and analyzed by mass spectrometry. (b) Striatal lysates from the intact (I) and lesioned (L) hemispheres were stained for a total protein stain (Ponceau S) and subsequently immunoblotted for tyrosine hydroxylase. The Uniprot ID for proteins detected in spinophilin immunoprecipitates that had a >18% decrease or increase in spectral counts isolated from either the extrasynaptic (c and d, respectively) or synaptic (e and f, respectively) fractions was inputted into the string database (http://www.string-db.org) to demonstrate connectivity between proteins. Proteins were assigned to categories based on protein function. The gene names for the proteins are used as identifiers. Mass spectrometry was performed on an Orbitrap Velos mass spectrometer.

To model PD, we unilaterally lesioned mouse substantia nigra using 6-hydroxydopamine (6-OHDA). This kills dopamine neurons that project from the nigra to the striatum. Striatal tissue from the contralateral, intact hemisphere and the ipsilateral, lesioned hemisphere was dissected, homogenized, fractionated, and immunoprecipitated as previously described [23, 24]. Spinophilin immunoprecipitates isolated from extrasynaptic (S2) or synaptic (S3) fractions were separated by SDS-PAGE, and gels were Coomassie stained. The gel regions containing spinophilin (Figure 1(a); red boxes) or associated proteins (Figure 1(a); black boxes) were excised, digested with trypsin, and processed by mass spectrometry to identify, de novo, spinophilin interacting proteins isolated from intact and lesioned striatum (see Section 2). We only used animals that had at least a 90% depletion of TH by immunoblotting (Figure 1(b)). There was little difference in total protein levels as evidenced by Ponceau staining (Figure 1(b)). Across 6 biological replicates (3 biological replicates analyzed on two separate days), we identified 125 total proteins that contained at least 48 spectral counts (an average of at least 2 spectral counts per sample across all 24 samples (lesion/intact; S2/S3); Table S1).

### 3.2. Spinophilin Interactions Are Modulated in Animal Models of PD

In order to understand how dopamine depletion may regulate spinophilin interactions on a global scale, we utilized state-of-the-art proteomic approaches to quantify proteins in the spinophilin immunoprecipitates in the striatum of intact or lesion animals. As a proof of this approach, we found that spinophilin had an increased association (~28%) with PP1 in the S3 fraction of the lesioned hemisphere. These results corroborate studies that we have previously performed in rats using immunoblotting approaches [8]. In addition to PP1, proteomic-based approaches allow us to identify changes without any a priori knowledge of the interaction. Using these approaches, to quantify spinophilin interactions, the total number of spinophilin-associated protein spectral counts from the intact and lesioned samples was normalized to the total number of spinophilin spectral counts in the same fraction and treatment. We then divided the normalized spectral count ratio of the lesion sample by that of the intact sample to generate a normalized lesion/intact ratio for each protein in the S2 and S3 fraction (Table S1). Using STRING database [27], we analyzed those proteins that had at least 50 spectral counts in either the S2 or S3 fraction and that had a normalized increase or decrease of 18%. This value was based on the decrease in NF-M protein isolated from spinophilin immunoprecipitates in lesion compared to intact striatum (see below). In the S3 fraction, 45 proteins had a decreased association with spinophilin, whereas 15 proteins had an increased association with spinophilin in the lesioned compared to intact hemisphere. In the S2 fraction, the number of increased and decreased associations was similar, 15 and 17 proteins, respectively. In both fractions, we observed decreases in the association of spinophilin with multiple cytoskeletal and synaptic signaling and scaffolding proteins in the lesioned compared to intact striatum (Figures 1() and 1(d)). Moreover, in the synaptic fraction of lesioned animals, we observed a decreased association of spinophilin with vesicle-trafficking proteins, ATPases, GTPases, and other protein classes (Figure 1(e)). In addition, we observed more myosin proteins and basement membrane proteins associating with spinophilin in the S3 fraction after lesioning (Figure 1(f)). In the S2 fraction, we observed an increase in the association of spinophilin with vesicle-trafficking proteins, such as clathrin, and cell adhesion proteins, such as contactin (Figure 1(d)).

### 3.3. NF-M Is Decreased in Spinophilin IPs after 6-OHDA Lesion

**Figure 2:**
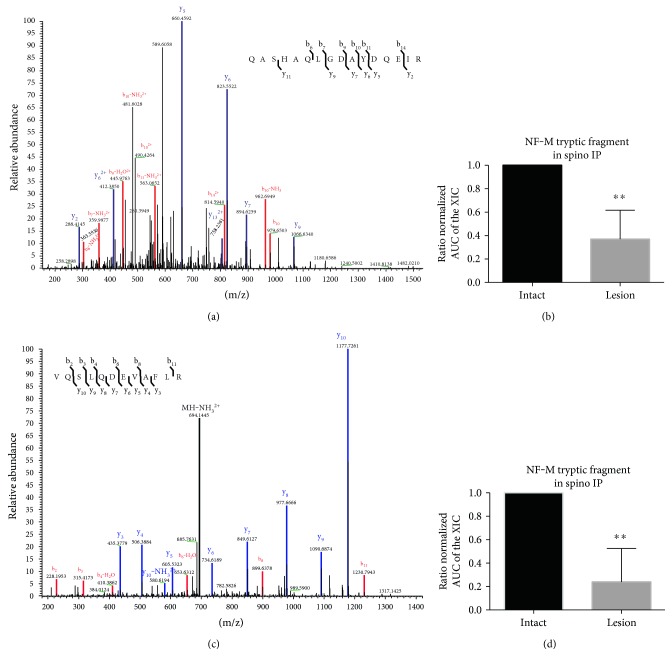
Reduced AUC of XICs matching NF-M in spinophilin immunoprecipitates isolated from the synaptic fraction of DA depleted striatum. MS/MS spectra matching the NF-M peptides QASHAQLGDAYDQEIR (a) and VQSLQDEVAFLR (c). The AUCs of the XIC matching the above peptides were normalized to relative levels (AUC of the XIC) of the average of 3 spinophilin peptides. These normalized values were divided by the corresponding value from the intact sample to generate a normalized ratio. The normalized ratios for QASHAQLGDAYDQEIR (b) and VQSLQDEVAFLR (d) are plotted. ^∗∗^*p* ≤ 0.01. *N* = 6/group. Mass spectrometry was performed on an Orbitrap Velos mass spectrometer.

Recent studies have found that neurofilament medium is localized to dendritic spines and is hypophosphorylated in the spines [20]. This makes it an interesting candidate for validation of the spectral counting data. Initial spectral counting data suggested that the spinophilin/NF-M interaction was decreased in the striatum of 6-OHDA-lesioned mice. To further confirm this decrease, the proteomic data from above were further analyzed. Specifically, the AUC of the XIC of two unique peptides (Figures 2(a) and 2(c)) on NF-M were quantified and normalized to the average AUC of three peptides matching spinophilin in the spinophilin IPs. We observed a decrease in NF-M in the spinophilin immunoprecipitates isolated from the striatum of the lesioned mice (Figures 2(b) and 2(d)^∗∗^*p* ≤ 0.01). This decrease was not due to any potential changes in spinophilin levels, as we normalized the AUC of the XIC to an average of the AUC of 3 spinophilin XICs. These data demonstrate that 6-OHDA lesion decreased the association of spinophilin with NF-M.

### 3.4. NF-M Coimmunoprecipitates with Spinophilin and PP1

**Figure 3:**
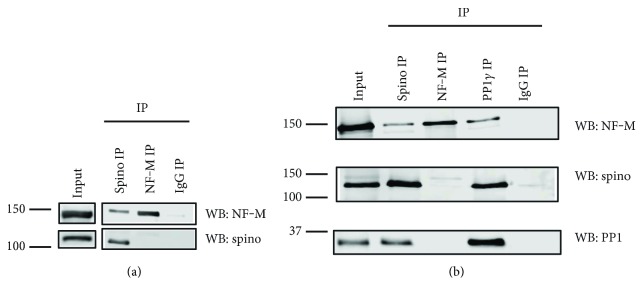
NF-M coimmunoprecipitates with spinophilin and PP1 in the mouse brain. Spinophilin (goat antibody), NF-M (mouse antibody), and the *γ*1 isoform of protein phosphatase 1 (PP1*γ*1; goat antibody) were immunoprecipitated from wild type mouse cortical (a) or striatal (b) tissue lysates homogenized in isotonic KCl buffer and immunoprecipitated with a goat antibody against spinophilin.

We previously found that NF-M was enriched in spinophilin immunoprecipitates isolated from wild type, but not knockout forebrain lysates using proteomic-based approaches [19]. To confirm specificity, we performed spinophilin immunoprecipitation and immunoblotted for NF-M isolated from mouse striatum. While NF-M and spinophilin associated in the striatum, we did not observe spinophilin in the NF-M IPs (Figures 3(a), 3(b)). Spinophilin [28, 29], but not NF-M [30], directly interacts with PP1. However, NF-M may associate with PP1 indirectly. Consistent with an association of PP1 with NF-M in an in vivo context, NF-M was detected in PP1 immunoprecipitates from the striatum (Figure 3(b)).

### 3.5. Nigral 6-Hydroxydopamine Lesion Increases Striatal Spinophilin Phosphorylation at Ser17 and Ser100

**Figure 4:**
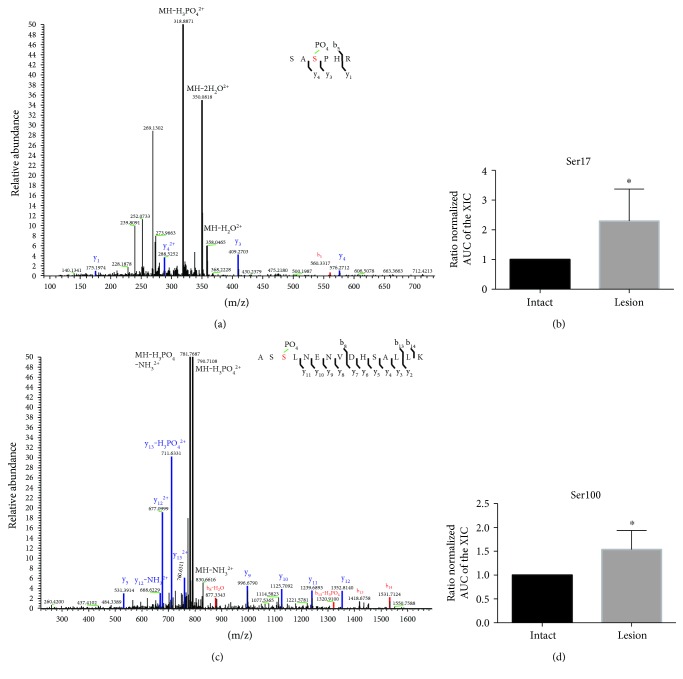
Synaptic spinophilin phosphorylation at Ser17 and Ser100 is enhanced by DA depletion. The substantia nigra of adult C57Bl6 mice was unilaterally lesioned with 6-OHDA. Striata from the intact and lesioned hemispheres were collected, fractionated into a membrane and synaptic fraction, and immunoprecipitated for spinophilin as in Figure 1. The region of the gel containing spinophilin and NF-M (Figure 1(a)) was excised and analyzed by mass spectrometry. (a) MS/MS spectra of a spinophilin tryptic fragment containing phosphorylated Ser17. (b) Quantification of the normalized AUC of the XIC matching the Ser17 in lesion and intact tissue normalized to the corresponding intact value. (c) MS/MS spectra of a spinophilin tryptic fragment containing phosphorylated Ser100. (d) Quantification of the normalized AUC of the XIC matching the Ser100 in lesion and intact tissue normalized to the corresponding intact value. ^∗^*p* ≤ 0.05 compared to the intact group. *N* = 6/group. Mass spectrometry was performed on an Orbitrap Velos mass spectrometer.

The mechanisms by which the association between spinophilin and synaptic proteins such as NF-M may be modulated following DA depletion are not known. Spinophilin phosphorylation has been shown to decrease spinophilin binding to F-actin and the alpha-2 adrenergic receptor [31–33]. Therefore, we evaluated spinophilin phosphorylation in our lesion and intact striatal spinophilin IPs. Using AUC of specific XICs that have MS/MS spectra that match spinophilin tryptic peptides phosphorylated at Ser17 and Ser100 (Figures 4(a) and 4(c), respectively), we found that spinophilin phosphorylation at both Ser17 and Ser100 was increased by 6-OHDA lesioning (Figures 4(b) and 4(d); ^∗^*p* ≤ 0.05). Therefore, enhanced kinase activity and subsequent increases in spinophilin phosphorylation are putative mechanism(s) for the observed decreased association of this scaffolding protein with multiple synaptic proteins, such as NF-M.

### 3.6. The Interaction of Spinophilin and NF-M in HEK293 Cells Requires Overexpression of the PKA Catalytic Subunit

**Figure 5:**
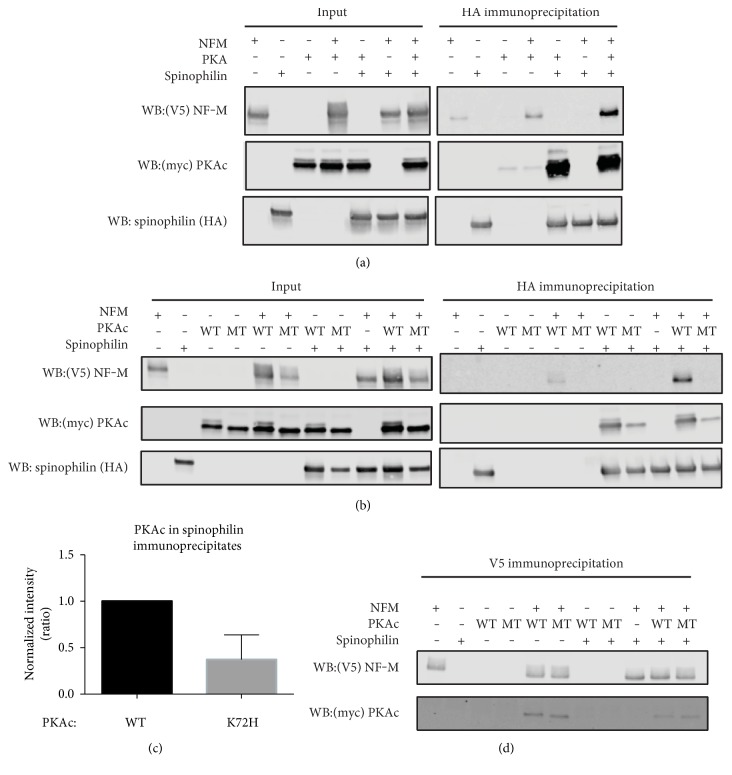
PKA overexpression is required for the spinophilin/NF-M interaction. (a) HEK293 cells were transfected with HA-tagged spinophilin, V5-tagged NF-M, and/or Myc-tagged PKAc. HA (goat antibody) immunoprecipitates were immunoblotted for HA (rabbit antibody), NF-M (mouse antibody), and Myc (mouse antibody). Data are representative of 3 independent experiments. (b, c) HEK293 cells were transfected with HA-tagged spinophilin, V5-tagged NF-M, and/or wild type or kinase dead (K72H mutant (MT)) myc-tagged PKAc. HA (goat antibody) (b) or V5 (rabbit antibody) (d) immunoprecipitates were immunoblotted for HA (rabbit antibody), NF-M (mouse antibody), and/or Myc (mouse antibody). (c) Decrease in the association of mutant compared to wild type PKA in the spinophilin immunoprecipitates (*p* = 0.0546, *n* = 3).

As spinophilin phosphorylation was regulated by DA depletion, we wanted to further determine if kinases can regulate the spinophilin/NF-M interaction. While there was little basal association between spinophilin and NF-M in a heterologous HEK293 cell line, upon overexpression of the catalytic subunit of PKA (PKAc), there was a robust and specific association between spinophilin and NF-M (Figure 5(a)). To determine if kinase activity was required for this interaction, we overexpressed a kinase dead mutant of PKA (K72H) [34]. There was no association of spinophilin with NF-M when the K72H mutant of PKA was expressed, indicating that this interaction was phosphorylation-dependent (Figure 5(b)). Spinophilin associated with both the wild type and the K72H PKAc (Figure 5(b)); however, there was an approximate 63% reduction in the association of spinophilin with the mutant PKAc (Figure 5(c); *N* = 3; *p* = 0.0546), suggesting that mutation of PKAc attenuates spinophilin binding to the kinase. Moreover, while PKAc was detected in the NF-M IPs (Figure 5(d)), the levels were low in some experiments and undetectable in others.

### 3.7. CDK5 Attenuates Spinophilin/NF-M Interaction

**Figure 6:**
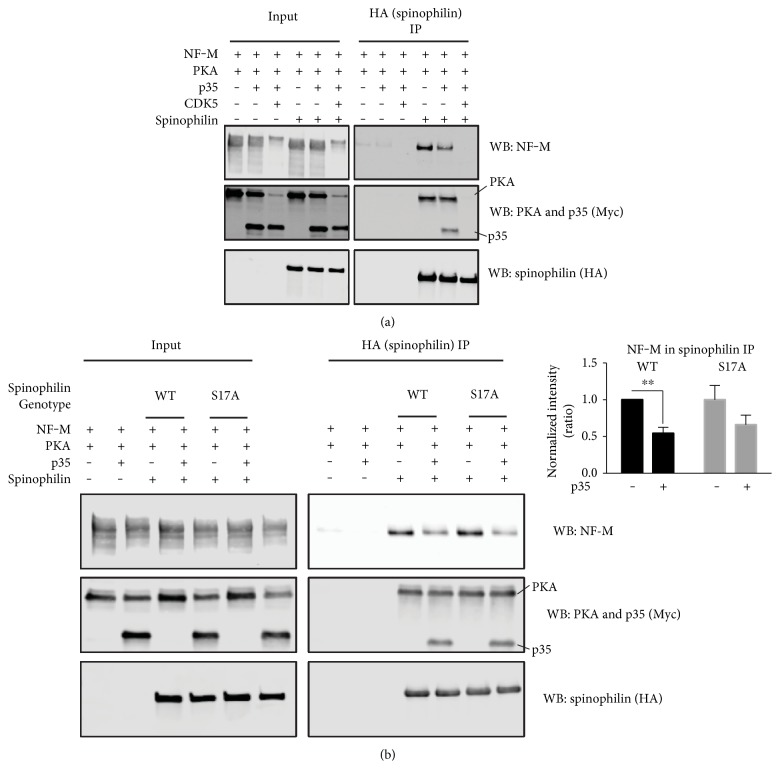
CDK5 activity attenuates the spinophilin/NF-M interaction. (a) HEK293 cells were transfected with spinophilin, NF-M, PKAc, p35, and/or CDK5. HEK293 cell lysates and HA (spinophilin) immunoprecipitates were immunoblotted for NF-M, Myc tag (PKA or p35), and HA. Data are representative of 3 independent experiments. (b) HEK293 cells were transfected with wild type or S17A spinophilin, NF-M, PKAc, and/or p35. HEK293 cell lysates and HA (spinophilin) immunoprecipitates were immunoblotted for NF-M, Myc tag (PKA or p35), and HA. A normalized intensity ratio was generated as described in methods. ^∗∗^*p* ≤ 0.01 compared to corresponding (i.e., same genotype) value obtained in the absence of p35 overexpression. *N* = 7‐8  per  group.

In addition to PKA, spinophilin is phosphorylated by CDK5 at Ser17. This phosphorylation was also enhanced in an animal model of PD (Figure 4(b)), further suggesting that CDK5 activity is increased by DA depletion [35, 36]. Using a heterologous cell model, we transfected spinophilin, NF-M, and PKAc in the absence or presence of CDK5 and its activator p35. Active CDK5 decreased the association between spinophilin and NF-M. This decrease occurred concurrently with decreased expression of NF-M and PKAc (Figure 6(a)). Overexpression of p35 alone decreased the association between spinophilin and NF-M without modulating NF-M or PKA expression (Figure 6(b); p35 expression ANOVA value *F*(1, 26) = 11.47; *P* = 0.0023). The p35-induced decreased association between spinophilin and NF-M was not due to spinophilin phosphorylation at Ser17, as mutation of this site had no effect on spinophilin binding to NF-M (Figure 6(b); genotype ANOVA value *F*(1,26) = 0.2494; *P* = 0.6217). Moreover, there was no interaction between genotype and p35 expression (*F*(1,26) = 0.2490; *P* = 0.6220). Post hoc analysis found a significant difference between the p35 conditions for the wild type spinophilin (*p* = 0.0086; *N* = 7‐8) and a trend for a decrease between these two conditions for the S17A mutant (*p* = 0.0587; *N* = 7‐8).

### 3.8. Spinophilin Regulates NF-M Phosphorylation State

**Figure 7:**
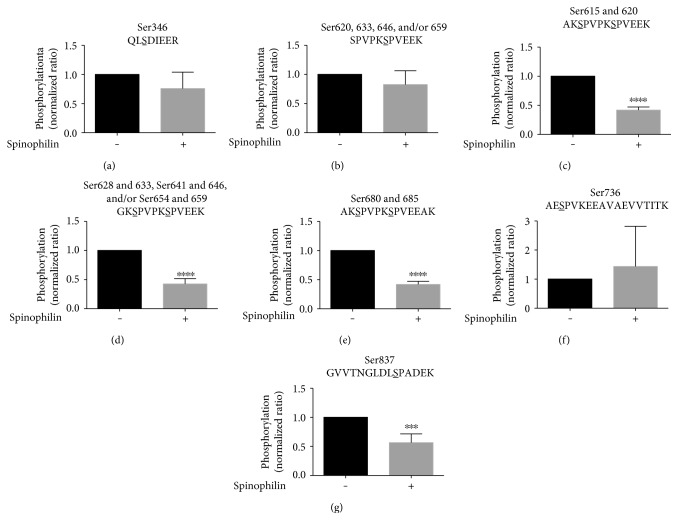
Spinophilin regulates NF-M phosphorylation. NF-M was overexpressed in HEK293 cells in the absence or presence of overexpressed spinophilin. V5 (rabbit antibody) immunoprecipitates were subjected to MS/MS analysis, and phosphorylation sites were quantified and normalized to the corresponding no spinophilin condition. (a–g) The ratios of phosphorylation for 7 specific phosphorylated tryptic peptides are shown. ^∗∗∗^*p* ≤ 0.001; ^∗∗∗∗^*p* ≤ 0.0001 compared to no spinophilin transfection. *N* = 5–7/group. Mass spectrometry was performed on a Q-exactive mass spectrometer.

To determine the putative implications of modulating the spinophilin/NF-M association, we evaluated the phosphorylation status of NF-M overexpressed in HEK293 cells in the absence or presence of overexpressed spinophilin. Using MS-based approaches, we detected NF-M tryptic peptides that had a single phosphorylation site at residues Ser346, Ser736, and Ser837. In addition, we detected phosphorylation of the second serine in the repeated peptide, SPVPKSPVEEK, corresponding to Ser620, Ser633, Ser646, and/or Ser659. In the KSP repeat domain, phosphorylation of the first serine on these repeat peptides may prevent cleavage of the lysine prior to the first serine. We detected doubly phosphorylated peptides matching the following tryptic peptides and residues: AKSPVPKSPVEEK, doubly phosphorylated at 615 and 620; GKSPVPKSPVEEK, doubly phosphorylated at Ser 628 and Ser633, Ser641 and Ser646, and/or Ser654 and Ser659; and AKSPVPKSPVEEAK, doubly phosphorylated at Ser 680 and Ser685. The MS/MS spectra were validated and hand annotated (Figures S1A–S1G). Phosphorylation of the peptides QLS(PO4)DIEER, SPVPKS(PO4)PVEEK, and AES(PO4)PVKEEAVAEVVTITK was unchanged by spinophilin overexpression (Figures 7(a), 7(b), and 7(f)); however, NF-M phosphorylation at Ser837 and at all of the doubly phosphorylated residues in the KSP repeat region was reduced by coexpression of spinophilin (Figures 7(c), 7(d), 7(e), and 7(g)). These studies were performed in the absence of overexpressed PKAc, suggesting that even though the association between spinophilin and NF-M is somewhat transient under these conditions, spinophilin can functionally modulate NF-M phosphorylation even in the absence of PKAc.

## 4. Discussion

### 4.1. 6-OHDA Lesioning Modulates Spinophilin Interactions and the Phosphorylation State of Spinophilin

Loss of striatal DA is associated with decreased dendritic spine density in the striatum, as well as loss of molecular correlates of learning and memory, such as LTD [1–5]. Spinophilin modulates dendritic spine density and regulates LTD [17, 37–39]. Spinophilin is known to associate with and modulate the function of multiple synaptic proteins [19, 26, 39–42]. We found that unilateral lesioning of the nigra of mice with 6-OHDA significantly increases phosphorylation of spinophilin at Ser17 and Ser100 in mouse striatum. Previous studies have demonstrated that spinophilin phosphorylation by PKA, CaMKII, and/or ERK attenuate its association with F-actin [31, 32, 43]. Concurrent with this altered spinophilin phosphorylation, we observed increases in the association of spinophilin with PP1, recapitulating what was previously found in rats [8]. While it may appear counterintuitive that greater association of spinophilin with PP1 reduces PP1 activity, previous studies have found that while spinophilin is important in targeting PP1 to various substrates, it also may inhibit its activity towards specific substrates [13]. This inhibition is substrate selective; therefore, tighter binding may affect some substrates but not others. Therefore, reduced PP1 activity in the context of greater kinase activity (such as CaMKII) [9, 10] may tip the balance towards phosphorylation. In addition to the increased association of spinophilin with PP1, we observed a decreased association of spinophilin with multiple synaptic proteins, including synaptic signaling and scaffolding proteins (e.g., GluN1 and SAPAP3) and cytoskeletal proteins (e.g., NF-M). Therefore, increased PP1 interaction with spinophilin along with decreased spinophilin association with synaptic proteins may both lead to decreased synaptic protein phosphorylation.

### 4.2. Mechanisms Regulating Spinophilin/NF-M Interaction

We detected NF-M in spinophilin immunoprecipitates isolated from striatal or forebrain lysates. We previously observed that this interaction was specific as NF-M had fewer spectral counts in spinophilin immunoprecipitates isolated from spinophilin knockout compared to wild type mice [19]. Moreover, we detected both spinophilin and NF-M in PP1 immunoprecipitates from striatal lysates; however, we did not detect spinophilin in NF-M immunoprecipitates. This may be due to occlusion of the NF-M antibody-binding site when spinophilin is bound, or it could be due to few spinophilin molecules bound to multiple NF-M molecules. In the latter scenario, when spinophilin is immunoprecipitated, it can extract multiple NF-M molecules, whereas immunoprecipitating multiple NF-M molecules would only extract a few (below the limit of detection) spinophilin molecules. When both spinophilin and NF-M were overexpressed in HEK293 cells in the presence of PKAc, there was a robust and specific interaction between the two proteins. As in the brain lysates, we only detected NF-M in the spinophilin immunoprecipitates and we did not detect spinophilin in the NF-M immunoprecipitates (data not shown). However, in the HEK293 cell experiments, we used tag antibodies, further validating the association and suggesting that antibody-site occlusion is not responsible for our inability to visualize spinophilin in NF-M immunoprecipitates, but rather, this may be due to few spinophilin molecules bound to multiple NF-M molecules, as described above. In order to determine if kinase activity was required for regulating the association between spinophilin and NF-M, we used a K72H kinase dead mutant of PKAc [34]. We found that the association between spinophilin and NF-M was not enhanced by this mutant form of PKAc. This finding suggests that catalytic activity of the kinase is required for the interaction between spinophilin and NF-M. However, an alternative interpretation may be that the mutant form of PKAc cannot act as a bridge between the two proteins. Interestingly, the association of spinophilin with the mutant PKA was decreased by 63% compared to spinophilin binding to the wild type PKAc. However, both wild type and K72H forms of PKAc did not consistently bind to NF-M. This lack of consistent binding to NF-M argues against the bridge hypothesis; however, we cannot completely rule out that PKAc overexpression may be acting as a bridge to stabilize the association between spinophilin and NF-M.

**Figure 8:**
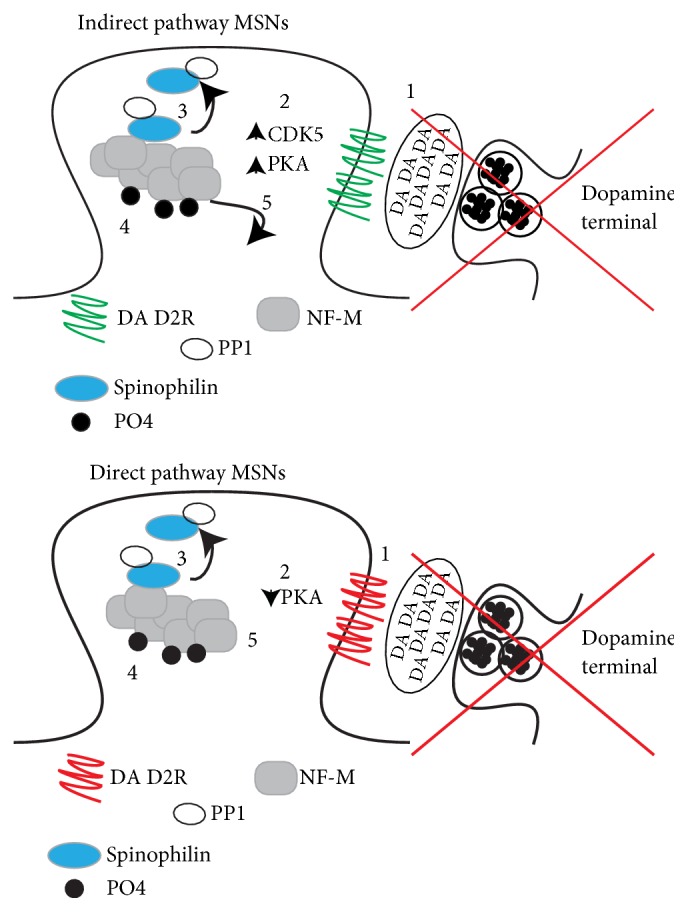
Hypothesis of how loss of DA may cell-specifically modulate the spinophilin/NF-M association and the putative in vivo functional consequences of altering this interaction. (1) Following loss of DA, DA D2 receptors (top) or D1 receptors (bottom) are no longer activated by DA. This leads to (2) increases in PKA and CDK5 activity in the indirect pathway MSNs (top) and decreases in PKA activity in the direct pathway MSNs (bottom). CDK5 activation overwhelms any increases in PKA activation observed in the indirect pathway MSNs. Both decreased PKA activity and increased CDK5 activation lead to (3) decreased association of spinophilin with NF-M and less PP1 targeting by spinophilin, which causes (4) increased phosphorylation of NF-M and (5) loss of NF-M in the dendritic spine.

Both spinophilin [32] and NF-M [44] are phosphorylated by PKA. Improper phosphorylation of neurofilaments can affect dendritic structure due to improper transport and cross-bridging of these proteins. Moreover, phosphorylation of neurofilament proteins can also impact their association with other proteins [45–47]. As stated above, we did not detect spinophilin in NF-M immunoprecipitates; therefore, it is also possible that PKA is enhancing the bridging of NF-M monomers and that there are more NF-M molecules associated with a single spinophilin molecule. Given the increased interaction between NF-M and spinophilin in HEK293 cells upon overexpression of PKA, increased phosphorylation of spinophilin and/or NF-M by PKA is most likely not contributing to the decreased interaction between spinophilin and NF-M that we observed in animal models of PD. We have utilized a heterologous cell system to explain changes in the spinophilin/NF-M interaction that are observed in an animal model of PD. It is important to reiterate that a heterologous cell system does not completely recapitulate what is observed in a neuron. Moreover, even the observed changes in dopamine-depleted animals have caveats. Specifically, it is important to note that our studies evaluate global changes in the spinophilin interactome. Given that the striatal MSNs are subdivided into direct and indirect pathway neurons and that the direct pathway neurons would be predicted to have less PKA-dependent activation (as DA D1 receptors activate PKA) upon loss of DA, there may be cell-specific differences in the activation of PKA. Previous studies have shown that NF-M interacts with the DA D1 receptor [20, 48]. This may suggest that NF-M localization may be different in the two MSN pathways; however, to our knowledge, this has not been tested. Therefore, spinophilin phosphorylation and/or interactions may be differentially modulated in the two MSN cell types (Figure 8()). Future studies will need to address if there are cell-specific dopamine depletion- and/or PKA activity-induced changes that contribute to the decreased association between spinophilin and NF-M.

In addition to increased phosphorylation of spinophilin at a PKA site in lesioned striatum, we observed augmented phosphorylation of spinophilin at a CDK5 site. Therefore, we next determined the functional role of CDK activity in modulating the association between spinophilin and NF-M in the context of PKA overexpression. Converse to the effect of PKA overexpression, overexpression of CDK5 and its activator, p35, decreased the association between spinophilin and NF-M as well as the expression of NF-M. CDK5-dependent regulation of NF-M expression is consistent with studies suggesting that downregulation of CDK5/p35 enhances neurofilament heavy expression [49]. It is important to note that expression of the neurofilament protein, neurofilament heavy, in the striatum is increased following short-term (3 days) 6-OHDA lesion of the nigra but is decreased following longer-term (14 days) depletion [50]. Moreover, CDK5 phosphorylates NF-M [51], and this was also apparent in our data, given a dramatic molecular weight shift in the NF-M band following CDK5 overexpression. Overexpression of the CDK5 activator, p35, alone had no obvious effects on NF-M expression; however, activation of endogenous CDK5 was sufficient to decrease the association between spinophilin and NF-M. While we observed increased phosphorylation of spinophilin at Ser17, a CDK5 site, in animal models of PD, it does not appear that increased phosphorylation at this site is responsible for the alterations in the spinophilin/NF-M association, as mutation of this site to an alanine did not rescue CDK5 activity-dependent decreases in the interaction between spinophilin and NF-M. Therefore, our data suggest that phosphorylation of NF-M, and/or an additional bridging protein, may be responsible for the CDK5-dependent decreases in the spinophilin/NF-M interaction. Unfortunately, NF-M is phosphorylated by CDK5 at multiple sites in the KSP repeat domain, making it difficult to determine if phosphorylation of NF-M is critical for regulating this interaction.

As stated above, spinophilin phosphorylation and interactions may be differentially regulated in the two MSN cell types. For instance, dopamine depletion using an MPTP model led to increases in CDK5 phosphorylation at a site that enhances CDK5 activity [36]. Interestingly, phosphorylation at this site is controlled by D2 DA receptor activity [36]. Therefore, taken together with the PKA data, we propose a model that changes in spinophilin phosphorylation and/or interactions may be different in the two MSN cell types. Moreover, given that there may be a greater effect of dopamine depletion on modulating indirect pathway MSNs [52, 53], these changes may predominate. Also, given that CDK5 activation, even in the presence of overexpressed PKA, led to dramatic reductions in the spinophilin/NF-M interaction, we speculate that CDK5 activity can attenuate any PKA-dependent increases that are observed. We propose a model whereby spinophilin phosphorylation and association with NF-M may be altered in the two MSN cell types by dopamine depletion (Figure 8()). It is currently unclear if some of the pathological changes, including loss of dendritic spines, occur only in the indirect pathway MSNs [52] or if the loss of spines occurs in both pathways [2, 4, 5, 54, 55]. However, it is known that signaling and other proteins have differential expression and/or function in the two MSN pathways [56–58].

### 4.3. Spinophilin Regulates NF-M Phosphorylation at Specific Residues: Implications in Neurodegeneration

Spinophilin is important in targeting PP1 to multiple substrates to decrease their phosphorylation. However, as stated above, spinophilin can also inhibit PP1 activity towards certain substrates [13]. Therefore, we wanted to determine the putative functional consequences of altering the spinophilin/NF-M association on NF-M phosphorylation. Overexpression of spinophilin in HEK293 cells reduced NF-M phosphorylation on multiple tryptic fragments. Previous mass spectrometry studies have shown that the KSP repeat region in bovine neurofilament proteins is highly phosphorylated [47]. Similar to spinophilin, NF-M is also located in dendritic spines. Moreover, levels of phosphorylated NF-M are lower in these synaptic locations compared to other regions [20]. Overexpression of spinophilin reduces NF-M phosphorylation in the KSP repeat domain in HEK293 cells. In addition to this region, spinophilin reduced NF-M phosphorylation at Ser837. Using a global proteomic analysis, previous reports show that Ser837 on NF-M is phosphorylated in human embryonic stem cells [59]. Moreover, Ser837 has been shown to be hyperphosphorylated in Alzheimer's disease postmortem samples [60]. Together, these data demonstrate that spinophilin can modulate NF-M phosphorylation in a heterologous cell system. We propose that mechanisms similar to this may be important in maintaining NF-M in a dephosphorylated state in the dendritic spine and future studies will need to delineate this role of spinophilin in vivo.

### 4.4. Conclusions

Understanding putative mechanisms by which NF-M phosphorylation in dendritic spines is regulated is of great interest, as dendritic spine density is decreased in PD [3–5] and these neurofilament proteins play a structural role in dendritic spines. Here, we demonstrate that spinophilin associates with NF-M in brain lysates. Furthermore, we show that in a heterologous cell system, this association is regulated by kinase activity/expression and that spinophilin overexpression reduces NF-M phosphorylation at several residues in the KSP repeat domain. Our data show that the NF-M/spinophilin interaction is decreased in an animal model of PD and that spinophilin can decrease NF-M phosphorylation. In addition to NF-M, our proteomic data have identified other synaptic spinophilin interacting proteins that are modulated by DA depletion, contributing to the hypothesis that spinophilin may be an important “hub” protein in PD [18]. Understanding how spinophilin modulates the phosphorylation status of these substrates in vivo and in specific cell types and how perturbations in spinophilin interactions contribute to pathological changes in striatal MSN spine density associated with PD will greatly enhance our understanding of the pathophysiology of this disorder.

## Supplementary Material

Supplementary Table (S1) Spectral counts of proteins containing at least 48 spectral counts across 6 biological replicates. The "Spectral Counts and Ratios" tab shows the spectral counts from each fraction and each replicate. The "Ratios Only" tab shows the total number of spectral counts from the S2 and S3 fraction for each protein across the 6 biological replicates. Both tabs also show a ratio that was calculated by dividing the number of spectral counts isolated from the lesioned hemisphere by the number of spectral counts isolated from the intact hemisphere. This calculation was performed independently for each subcellular fraction. Supplemental Figure S1. Sample MS/MS spectra matching NF-M phosphopeptides that were detected in the NF-M immunoprecipitates isolated from HEK293 cells. Supplemental Figure S2. NF-M sequence in the KSP-repeat domain with tryptic peptides that were identified in the NF-M immunoprecipitates isolated from HEK293 cells.

## Abbreviations

6-OHDA: 6-Hydroxydopamine
AUC: Area under the curve
CDK5: Cyclin-dependent protein kinase 5
D1R: Dopamine D1 receptor
D2R: Dopamine D2 receptor
DA: Dopamine
HEK293: Human embryonic kidney 293FT cells
MSN: Medium spiny neuron
NF-M: Neurofilament medium
PD: Parkinson disease
PKA: Protein kinase A
PKAc: PKA catalytic subunit
PP1: Protein phosphatase 1
PSD: Postsynaptic density
XIC: Extracted ion chromatogram.
