# Supplementary material for: Mechanisms and Consequences of Dopamine Depletion-Induced Attenuation of the Spinophilin/Neurofilament Medium Interaction
Source: Neural Plast. 2017 May 28;2017:4153076. doi: 10.1155/2017/4153076 (PMC5467389; doi:10.1155/2017/4153076)
Supplement: Supplementary file 1 — Supplementary Table (S1) Spectral counts of proteins containing at least 48 spectral counts across 6 biological replicates. The "Spectral Counts and Ratios" tab shows the spectral counts from each fraction and each replicate. The "Ratios Only" tab shows the total number of spectral counts from the S2 and S3 fraction for each protein across the 6 biological replicates. Both tabs also show a ratio that was calculated by dividing the number of spectral counts isolated from the lesioned hemisphere by the number of spectral counts isolated from the intact hemisphere. This calculation was performed independently for each subcellular fraction. Supplemental Figure S1. Sample MS/MS spectra matching NF-M phosphopeptides that were detected in the NF-M immunoprecipitates isolated from HEK293 cells. Supplemental Figure S2. NF-M sequence in the KSP-repeat domain with tryptic peptides that were identified in the NF-M immunoprecipitates isolated from HEK293 cells. [file 4153076.f1.pdf]

Supplementary Table (S1) Legend

Spectral counts of proteins containing at least 48 spectral counts across 6 biological replicates. The "Spectral Counts and Ratios" tab shows the spectral counts from each fraction and each replicate. The "Ratios Only" tab shows the total number of spectral counts from the S2 and S3 fraction for each protein across the 6 biological replicates. Both tabs also show a ratio that was calculated by dividing the number of spectral counts isolated from the lesioned hemisphere by the number of spectral counts isolated from the intact hemisphere. This calculation was performed independently for each subcellular fraction.

Supplemental Figure S1. Sample MS/MS spectra matching NF-M phosphopeptides that were detected in the NF-M immunoprecipitates isolated from HEK293 cells.

Supplemental Figure S2. NF-M sequence in the KSP-repeat domain with tryptic peptides that were identified in the NF-M immunoprecipitates isolated from HEK293 cells.

		Run 1												Run 2															S2 Lesion/Intact	S3 Lesion/Intact	Average Spectral counts from specific fraction were divided by spinophilin spectral counts in the same fraction and treatment. The Lesion values were then divided by the intact values
Name	Accession Number	S2 Intact			S2 Lesion			S3 Intact			S3 Lesion			S2 Intact			S2 Lesion			S3 Intact			S3 Lesion			S2 Spectral Counts	S3 Spectral Counts	Total	Normalized Ratio	Normalized Ratio	
SRC kinase signaling inhibitor 1 OS=Mus musculus GN=Srcin1 PE=1 SV=2	Q9QWI6	278	278	214	342	281	372	16	3	27	47	24	16	435	365	375	371	346	373	95	141	60	55	73	73	4030	630	4660	1.10	0.85	
Clathrin heavy chain 1 OS=Mus musculus GN=Cltc PE=1 SV=3	Q68FD5	55	51	36	56	25	26	114	2	21	5	1	2	139	149	91	208	215	314	5	7	1	2	6	6	1365	172	1537	1.66	0.15	
Ras GTPase-activating protein SynGAP OS=Mus musculus GN=Syngap1 PE=3 SV=2	F6SEU4	54	39	52	83	35	44	63	47	72	106	101	72	144	168	178	143	113	93	186	199	59	86	97	107	1146	1195	2341	0.82	0.91	
Breakpoint cluster region protein OS=Mus musculus GN=Bcr PE=1 SV=3	Q6PAJ1	26	34	13	26	23	34	0	0	0	0	0	0	189	144	159	166	151	146	0	0	1	0	0	0	1111	1	1112	0.99	0.00	
Neurabin-2 OS=Mus musculus GN=Ppp1r9b PE=1 SV=1	Q6R891	89	91	50	77	80	99	338	271	263	316	389	241	109	104	118	110	107	76	261	245	62	159	155	172	1110	2872	3982	1.00	1.00	
Neurofilament medium polypeptide OS=Mus musculus GN=Nefm PE=1 SV=4	P08553	11	16	43	25	48	36	178	73	114	97	60	70	141	129	164	172	88	87	264	347	56	184	259	172	960	1874	2834	0.92	0.82	
Protein phosphatase 1 regulatory subunit 12A OS=Mus musculus GN=Ppp1r12a PE=1 SV=2	Q9DBR7	45	51	33	57	47	67	3	0	0	0	0	0	97	94	71	72	99	83	6	10	9	2	2	2	816	34	850	1.11	0.22	
ERC protein 2 OS=Mus musculus GN=Erc2 PE=1 SV=2	Q6PH08	74	85	52	66	63	113	82	23	45	49	53	44	50	50	71	57	41	53	138	116	24	84	84	83	775	825	1600	1.05	0.93	
Myosin-10 OS=Mus musculus GN=Myh10 PE=1 SV=2	Q61879	106	86	51	86	69	65	20	13	17	13	12	8	39	34	24	42	44	24	20	27	12	13	28	32	670	215	885	0.99	0.98	
Drebrin OS=Mus musculus GN=Dbn1 PE=1 SV=4	Q9QXS6	31	51	18	34	33	54	47	14	1	4	5	0	83	64	81	80	58	55	30	42	13	19	21	22	642	218	860	0.98	0.49	
Spectrin alpha chain, brain OS=Mus musculus GN=Sptan1 PE=1 SV=4	P16546	64	51	43	55	51	41	113	8	34	14	7	6	38	41	66	78	44	21	50	78	28	31	60	62	593	491	1084	0.98	0.58	
Actin, cytoplasmic 1 OS=Mus musculus GN=Actb PE=1 SV=1	P60710	53	54	40	59	44	40	32	36	30	30	33	28	38	36	33	44	31	29	31	35	26	84	35	27	501	427	928	0.99	1.25	
Protein bassoon OS=Mus musculus GN=Bsn PE=1 SV=4	O88737	66	52	37	65	63	63	2	0	3	1	1	0	19	25	11	10	30	11	1	0	1	0	1	2	452	12	464	1.18	0.72	
Alpha-actinin-1 OS=Mus musculus GN=Actn1 PE=1 SV=1	Q7TPR4	44	84	48	51	88	61	62	8	4	7	1	0	10	13	13	13	8	4	2	7	3	6	5	6	437	111	548	1.08	0.29	
Glutamate [NMDA] receptor subunit zeta-1 OS=Mus musculus GN=Grin1 PE=1 SV=1	P35438	35	33	26	31	22	22	18	17	15	22	16	17	51	42	61	49	30	31	42	47	17	13	17	27	433	268	701	0.76	0.72	
Disks large homolog 4 OS=Mus musculus GN=Dlg4 PE=1 SV=1	Q62108	44	59	50	66	96	59	87	108	70	118	117	58	12	9	12	11	10	3	11	23	16	11	13	17	431	649	1080	1.35	1.07	
Desmoplakin OS=Mus musculus GN=Dsp PE=3 SV=1	E9Q557	24	31	56	17	18	21	14	30	39	42	33	55	25	54	44	53	52	35	11	9	19	0	2	4	430	258	688	0.86	1.12	
Unconventional myosin-Va OS=Mus musculus GN=Myo5a PE=1 SV=2	Q99104	64	43	29	44	44	35	19	11	19	12	16	5	34	24	18	26	23	9	12	21	6	19	14	15	393	169	562	0.87	0.93	
Contactin-1 OS=Mus musculus GN=Cntn1 PE=1 SV=1	P12960	19	25	15	29	21	32	78	1	0	0	0	0	20	25	45	53	19	36	4	20	10	12	6	1	339	132	471	1.30	0.17	
IQ motif and SEC7 domain-containing protein 1 OS=Mus musculus GN=Iqsec1 PE=1 SV=2	Q8R0S2	12	18	14	24	5	28	12	0	1	5	0	2	36	47	30	41	45	38	22	41	15	11	28	12	338	149	487	1.18	0.64	
Tubulin alpha-1A chain OS=Mus musculus GN=Tuba1a PE=1 SV=1	P68369	11	11	13	11	8	14	18	8	12	10	11	7	45	35	32	43	36	56	24	26	6	28	12	0	315	162	477	1.17	0.73	
Glycogen phosphorylase, muscle form OS=Mus musculus GN=Pygm PE=1 SV=3	Q9WUB3	41	53	42	56	55	66	33	72	46	63	55	33	0	0	0	0	0	0	0	0	0	0	0	0	313	302	615	1.33	1.01	
ATP-dependent RNA helicase A OS=Mus musculus GN=Dhx9 PE=1 SV=2	O70133	1	2	2	5	2	2	0	0	0	2	11	5	39	43	57	69	29	48	0	17	12	0	6	5	299	58	357	1.10	1.01	
LisH domain and HEAT repeat-containing protein KIAA1468 OS=Mus musculus GN=Kiaa1468 PE=1 SV=1	Q148V7	30	41	15	39	33	50	1	0	0	0	1	2	20	13	5	12	10	23	0	0	1	1	1	1	291	7	298	1.38	6.03	
Junction plakoglobin OS=Mus musculus GN=Jup PE=1 SV=3	Q02257	21	17	37	14	15	13	8	16	21	14	13	25	14	37	44	32	24	14	10	3	6	0	1	1	282	118	400	0.67	0.85	
Arf-GAP with GTPase, ANK repeat and PH domain-containing protein 2 OS=Mus musculus GN=Agap2 PE=1 SV=1	Q3UHD9	7	7	0	21	0	7	0	0	2	2	1	1	35	40	27	33	40	53	19	23	7	4	6	2	270	67	337	1.36	0.32	
Unconventional myosin-VI OS=Mus musculus GN=Myo6 PE=1 SV=1	Q64331	2	1	1	2	1	1	1	0	0	0	0	0	48	32	49	71	35	27	1	3	11	0	0	6	270	22	292	1.05	0.38	
Matrin-3 OS=Mus musculus GN=Matr3 PE=1 SV=1	Q8K310	4	16	9	7	22	26	4	0	0	0	4	0	28	24	37	45	13	29	7	3	11	5	5	7	260	46	306	1.23	0.84	
Spectrin beta chain, brain 1 OS=Mus musculus GN=Sptbn1 PE=1 SV=2	Q62261	30	31	19	26	22	17	35	1	13	6	1	0	15	17	21	28	23	7	16	34	10	12	34	35	256	197	453	0.95	0.81	
Alpha-actinin-2 OS=Mus musculus GN=Actn2 PE=1 SV=1	Q9JI91	38	54	33	43	30	21	0	0	0	0	0	0	6	4	7	5	6	2	1	9	3	0	5	5	249	23	272	0.77	0.77	
Neural cell adhesion molecule 1 OS=Mus musculus GN=Ncam1 PE=1 SV=3	P13595	15	17	19	15	8	18	39	1	6	1	1	1	25	23	29	31	22	24	27	36	8	16	20	14	246	170	416	0.94	0.46	
Heterogeneous nuclear ribonucleoprotein U OS=Mus musculus GN=Hnrnpu PE=1 SV=1	Q8VEK3	8	21	20	14	18	23	50	14	33	34	21	33	19	21	33	29	11	27	54	62	24	42	33	40	244	440	684	1.02	0.86	
V-type proton ATPase 116 kDa subunit a isoform 1 OS=Mus musculus GN=Atp6v0a1 PE=1 SV=3	Q9Z1G4	24	31	28	38	11	20	85	7	16	1	0	1	16	14	16	19	6	9	5	9	1	0	1	0	232	126	358	0.82	0.02	
Sodium/potassium-transporting ATPase subunit alpha-3 OS=Mus musculus GN=Atp1a3 PE=1 SV=1	Q6PIC6	51	35	29	27	22	55	531	160	118	18	3	1	2	1	0	0	2	0	1	1	0	1	0	0	224	834	1058	0.92	0.03	
AP-2 complex subunit alpha-1 OS=Mus musculus GN=Ap2a1 PE=1 SV=1	P17426	34	54	30	35	7	11	114	3	21	0	0	3	5	4	9	6	0	4	4	7	1	3	1	0	199	157	356	0.47	0.05	
Rap guanine nucleotide exchange factor 4 OS=Mus musculus GN=Rapgef4 PE=1 SV=1	Q9EQZ6	31	40	23	29	29	38	7	0	1	3	3	0	0	1	2	1	1	0	0	1	0	5	0	1	195	21	216	1.03	1.34	
Myelin basic protein OS=Mus musculus GN=Mbp PE=1 SV=2	P04370	13	13	17	19	11	18	7	5	13	15	10	9	20	12	13	15	12	24	5	6	4	4	4	3	187	85	272	1.15	1.13	
Heat shock cognate 71 kDa protein OS=Mus musculus GN=Hspa8 PE=1 SV=1	P63017	24	25	15	21	17	16	25	7	16	5	4	3	6	15	12	10	12	8	8	10	2	4	7	4	181	95	276	0.88	0.40	
Synaptopodin OS=Mus musculus GN=Synpo PE=1 SV=2	Q8CC35	28	41	26	28	21	13	2	0	0	0	0	0	4	2	5	4	2	1	0	3	1	0	1	1	175	8	183	0.67	0.34	
Neurofilament light polypeptide OS=Mus musculus GN=Nefl PE=1 SV=5	P08551	13	13	14	10	12	9	21	15	29	23	18	13	15	18	17	22	16	7	26	50	17	60	34	27	166	333	499	0.86	1.11	
Catenin alpha-2 OS=Mus musculus GN=Ctnna2 PE=1 SV=3	Q61301	17	31	19	22	36	24	41	0	0	0	0	0	3	3	1	2	2	1	0	1	0	0	1	1	161	44	205	1.20	0.05	
Alpha-internexin OS=Mus musculus GN=Ina PE=1 SV=2	P46660	5	7	5	11	12	11	15	17	14	9	12	8	24	16	14	21	18	11	27	48	23	85	37	23	155	318	473	1.21	1.22	
Disks large homolog 2 OS=Mus musculus GN=Dlg2 PE=1 SV=1	Q91XM9	21	25	22	23	26	21	32	33	32	37	55	34	4	6	3	0	2	0	23	32	12	29	20	15	153	354	507	0.91	1.17	
SH3 and multiple ankyrin repeat domains protein 3 OS=Mus musculus GN=Shank3 PE=1 SV=2	Q4ACU6	11	10	5	17	4	2	11	14	15	17	9	5	17	11	30	25	12	9	19	24	18	4	17	23	153	176	329	0.84	0.75	
Myosin-9 OS=Mus musculus GN=Myh9 PE=1 SV=4	Q8VDD5	22	22	10	21	15	13	19	18	14	22	23	15	13	6	4	12	9	2	18	23	15	12	29	31	149	239	388	0.96	1.24	
2',3'-cyclic-nucleotide 3'-phosphodiesterase OS=Mus musculus GN=Cnp PE=1 SV=3	P16330	16	15	12	13	13	8	32	15	19	12	6	7	16	11	10	11	13	9	13	18	5	7	14	10	147	158	305	0.86	0.55	
Dihydropyrimidinase-related protein 2 OS=Mus musculus GN=Dpysl2 PE=1 SV=2	O08553	21	18	10	19	15	15	27	8	19	6	5	7	6	9	7	6	9	6	0	3	2	0	3	0	141	80	221	1.01	0.36	
Serine/threonine-protein phosphatase PP1-alpha catalytic subunit OS=Mus musculus GN=Ppp1ca PE=1 SV=1	P62137	15	15	7	15	11	10	26	25	28	24	31	23	13	11	12	11	13	6	17	18	16	44	23	21	139	296	435	0.92	1.28	
cAMP and cAMP-inhibited cGMP 3',5'-cyclic phosphodiesterase 10A OS=Mus musculus GN=Pde10a PE=1 SV=2	Q8CA95	17	33	15	23	13	22	2	0	0	0	0	0	1	4	2	2	2	2	0	0	0	0	0	0	137	2	139	0.90	0.00	
Protein piccolo OS=Mus musculus GN=Pclo PE=1 SV=3	Q9QYX7	15	9	11	26	20	20	2	0	4	2	2	1	7	10	4	5	5	2	3	1	1	0	4	3	134	23	157	1.42	1.10	
Disks large-associated protein 3 OS=Mus musculus GN=Dlgap3 PE=1 SV=1	Q6PFD5	18	13	15	18	0	2	20	21	15	20	13	15	7	11	28	11	6	2	43	40	11	23	25	23	131	269	400	0.43	0.80	
Calcium-binding mitochondrial carrier protein Aralar1 OS=Mus musculus GN=Slc25a12 PE=1 SV=1	Q8BH59	13	13	11	10	8	10	18	0	0	0	0	0	12	14	5	9	14	12	1	1	0	0	0	0	131	20	151	0.95	0.00	
AP-2 complex subunit beta OS=Mus musculus GN=Ap2b1 PE=1 SV=1	Q9DBG3	13	23	14	18	22	20	156	10	16	1	4	1	5	3	3	4	3	2	4	3	1	2	2	2	130	202	332	1.16	0.06	
A-kinase anchor protein 5 OS=Mus musculus GN=Akap5 PE=3 SV=2	D3YVF0	1	1	0	2	1	0	3	4	3	20	9	2	29	14	24	28	10	19	0	0	1	2	0	1	129	45	174	0.89	3.11	
Gap junction alpha-1 protein OS=Mus musculus GN=Gja1 PE=1 SV=2	P23242	19	14	13	16	15	14	15	17	19	17	22	14	10	5	4	6	9	3	6	13	5	16	6	7	128	157	285	0.99	1.10	
Microtubule-associated protein 6 OS=Mus musculus GN=Map6 PE=1 SV=2	Q7TSJ2	0	6	3	6	0	1	47	0	2	0	0	0	8	18	16	16	17	31	3	4	4	1	0	0	122	61	183	1.42	0.02	
Sickle tail protein OS=Mus musculus GN=Skt PE=1 SV=1	A2AQ25	3	4	3	7	5	5	0	0	0	0	0	0	19	13	16	12	13	17	0	0	4	0	0	0	117	4	121	1.04	0.00	
Mitogen-activated protein kinase kinase kinase 5 OS=Mus musculus GN=Map3k5 PE=1 SV=3	O35099	1	0	0	1	2	1	0	0	0	0	0	0	20	21	12	20	18	18	0	0	0	0	0	0	114	0	114	1.14	#DIV/0!	
Calcium/calmodulin-dependent protein kinase type II subunit beta OS=Mus musculus GN=Camk2b PE=1 SV=2	P28652	14	15	9	14	11	8	19	10	12	6	12	3	11	11	4	5	8	3	11	14	4	6	10	3	113	110	223	0.78	0.57	
Disks large-associated protein 2 OS=Mus musculus GN=Dlgap2 PE=1 SV=2	Q8BJ42	0	3	0	0	0	4	4	4	1	11	4	0	14	19	23	17	7	12	27	30	20	5	8	12	99	126	225	0.69	0.47	
Valine--tRNA ligase OS=Mus musculus GN=Vars PE=2 SV=1	Q9Z1Q9	0	0	0	5	18	26	0	0	0	0	0	0	7	15	4	5	8	10	0	0	0	0	0	0	98	0	98	2.83	#DIV/0!	
Guanine nucleotide-binding protein G(o) subunit alpha OS=Mus musculus GN=Gnao1 PE=1 SV=3	P18872	11	7	8	8	8	7	16	5	7	3	2	3	9	8	7	9	7	5	5	10	2	6	5	4	94	68	162	0.90	0.51	
Myosin-4 OS=Mus musculus GN=Myh4 PE=1 SV=1	Q5SX39	15	9	12	21	16	14	73	11	21	14	87	7	3	0	0	0	0	0	0	0	0	0	0	0	90	213	303	1.34	1.03	
F-box only protein 41 OS=Mus musculus GN=Fbxo41 PE=1 SV=3	Q6NS60	13	20	11	14	3	14	11	1	2	12	14	4	3	1	2	3	0	5	5	3	5	15	13	4	89	89	178	0.80	2.31	
Brain-specific angiogenesis inhibitor 1-associated protein 2 OS=Mus musculus GN=Baiap2 PE=1 SV=2	Q8BKX1	2	2	0	3	3	2	0	0	0	0	0	0	13	9	13	18	11	7	3	9	3	0	5	3	83	23	106	1.15	0.54	
Calcium/calmodulin-dependent protein kinase type II subunit alpha OS=Mus musculus GN=Camk2a PE=1 SV=2	P11798	3	2	1	1	0	0	5	0	2	1	1	1	14	17	8	10	17	7	21	33	10	43	16	9	80	142	222	0.79	1.01	
MTSS1-like protein OS=Mus musculus GN=Mtss1l PE=1 SV=1	Q6P9S0	12	22	7	7	20	9	0	0	0	0	0	0	2	0	0	0	0	0	0	0	0	0	0	0	79	0	79	0.86	#DIV/0!	
Tubulin beta-5 chain OS=Mus musculus GN=Tubb5 PE=1 SV=1	P99024	10	8	9	10	6	9	16	2	10	5	4	2	6	0	2	3	2	12	6	6	3	0	2	1	77	57	134	1.23	0.33	
Copine-5 OS=Mus musculus GN=Cpne5 PE=2 SV=1	Q8JZW4	10	8	6	9	4	6	8	5	8	6	1	3	5	6	6	8	5	3	5	8	4	4	4	2	76	58	134	0.87	0.53	
Transcriptional activator protein Pur-alpha OS=Mus musculus GN=Pura PE=1 SV=1	P42669	11	9	3	9	10	8	5	0	1	0	0	0	5	4	3	2	6	2	0	0	0	0	0	0	72	6	78	1.08	0.00	
Regulator of nonsense transcripts 1 OS=Mus musculus GN=Upf1 PE=1 SV=2	Q9EPU0	4	10	3	10	0	3	0	0	0	0	0	0	7	8	7	5	3	11	0	0	0	0	0	0	71	0	71	0.84	#DIV/0!	
Tubulin beta-4B chain OS=Mus musculus GN=Tubb4b PE=1 SV=1	P68372	0	1	1	2	1	1	2	0	1	2	1	0	15	9	8	6	13	12	10	12	2	0	7	6	69	43	112	1.05	0.60	
Oligodendrocyte-myelin glycoprotein OS=Mus musculus GN=Omg PE=1 SV=1	Q63912	4	10	4	8	10	7	0	0	0	0	0	0	3	4	4	6	1	1	2	4	1	0	0	0	62	7	69	1.16	0.00	
Histone H1.3 OS=Mus musculus GN=Hist1h1d PE=1 SV=2	P43277	2	4	3	3	3	3	9	9	14	21	23	20	8	2	10	14	6	3	26	24	24	33	22	18	61	243	304	1.13	1.30	
ATP synthase subunit gamma, mitochondrial OS=Mus musculus GN=Atp5c1 PE=1 SV=1	Q91VR2	7	8	6	6	6	7	6	2	3	0	0	0	6	3	2	3	4	3	0	0	0	0	0	0	61	11	72	0.93	0.00	
Guanine nucleotide-binding protein G(z) subunit alpha OS=Mus musculus GN=Gnaz PE=2 SV=4	O70443	7	6	3	5	2	3	2	0	0	0	0	0	6	7	4	7	7	3	0	1	0	0	0	0	60	3	63	0.84	0.00	
Unconventional myosin-Ib OS=Mus musculus GN=Myo1b PE=2 SV=3	P46735	1	1	0	1	1	2	6	0	0	6	0	0	10	7	15	14	5	2	18	21	13	10	14	16	59	104	163	0.75	0.80	
SRSF protein kinase 2 OS=Mus musculus GN=Srpk2 PE=1 SV=2	O54781	4	5	4	5	12	21	0	0	0	0	0	0	1	3	2	0	1	1	0	0	0	0	0	0	59	0	59	2.15	#DIV/0!	
ATPase family AAA domain-containing protein 3 OS=Mus musculus GN=Atad3 PE=1 SV=1	Q925I1	5	8	4	6	5	7	0	0	0	0	0	0	3	5	2	3	6	3	0	0	0	0	0	0	57	0	57	1.14	#DIV/0!	
60S acidic ribosomal protein P0 OS=Mus musculus GN=Rplp0 PE=1 SV=3	P14869	7	5	3	7	7	7	2	0	0	0	2	0	4	4	2	1	6	3	1	1	0	0	0	0	56	6	62	1.27	0.50	
Calcium-activated potassium channel subunit alpha-1 OS=Mus musculus GN=Kcnma1 PE=1 SV=2	Q08460	0	7	3	5	6	16	6	0	0	0	0	0	2	4	1	3	4	2	0	0	3	0	0	0	53	9	62	2.16	0.00	
Tubulin alpha-1B chain OS=Mus musculus GN=Tuba1b PE=1 SV=2	P05213	3	9	7	4	0	11	30	11	10	23	12	6	3	4	2	3	1	3	3	2	0	0	0	0	50	97	147	0.80	0.74	
Glycogen phosphorylase, muscle form OS=Mus musculus GN=Pygm PE=1 SV=3	Q9WUB3	11	10	6	8	7	8	12	8	13	6	9	4	0	0	0	0	0	0	0	0	0	0	0	0	50	52	102	0.87	0.58	
Annexin A2 OS=Mus musculus GN=Anxa2 PE=1 SV=2	P07356	3	5	10	1	4	0	4	5	4	6	5	7	2	6	5	5	7	2	1	2	5	0	2	1	50	42	92	0.63	1.01	
Guanine nucleotide-binding protein G(I)/G(S)/G(T) subunit beta-1 OS=Mus musculus GN=Gnb1 PE=1 SV=3	P62874	4	5	4	6	5	2	6	2	2	0	1	0	4	4	3	5	5	2	0	2	0	0	0	0	49	13	62	1.06	0.08	
Homer protein homolog 1 OS=Mus musculus GN=Homer1 PE=1 SV=2	Q9Z2Y3	6	2	5	3	2	0	7	1	5	5	1	3	7	3	6	5	5	1	8	14	8	13	10	11	45	86	131	0.56	1.01	
AP-2 complex subunit alpha-2 OS=Mus musculus GN=Ap2a2 PE=1 SV=2	P17427	5	20	5	10	0	0	58	0	2	0	0	0	2	0	0	1	0	0	0	0	0	0	0	0	43	60	103	0.35	0.00	
Desmoglein-1-alpha OS=Mus musculus GN=Dsg1a PE=2 SV=2	Q61495	2	3	1	1	2	1	2	2	2	2	3	4	2	6	9	7	4	2	0	0	1	0	0	0	40	16	56	0.76	1.29	
Centromere protein V OS=Mus musculus GN=Cenpv PE=2 SV=2	Q9CXS4	2	5	6	3	5	3	2	1	1	0	0	0	2	2	3	4	2	0	3	7	3	3	4	4	37	28	65	0.87	0.65	
Protein Dok-7 OS=Mus musculus GN=Dok7 PE=1 SV=1	Q18PE0	5	10	5	7	5	4	4	5	8	5	5	6	0	0	0	0	0	0	0	0	0	0	0	0	36	33	69	0.82	0.95	
Tubulin beta-2A chain OS=Mus musculus GN=Tubb2a PE=1 SV=1	Q7TMM9	1	1	3	5	1	1	8	1	1	2	1	0	5	3	3	3	3	7	1	4	1	0	2	1	36	22	58	1.28	0.38	
Kinesin-like protein KIF2A OS=Mus musculus GN=Kif2a PE=1 SV=2	P28740	8	12	6	8	0	1	15	0	2	0	1	1	0	0	0	0	0	0	0	0	0	0	0	0	35	19	54	0.35	0.12	
Tubulin alpha-4A chain OS=Mus musculus GN=Tuba4a PE=1 SV=1	P68368	1	1	1	2	3	2	2	0	2	1	1	1	4	4	3	4	4	3	2	3	0	1	2	2	32	17	49	1.31	0.89	
Glyceraldehyde-3-phosphate dehydrogenase OS=Mus musculus GN=Gapdh PE=1 SV=2	P16858	6	4	3	8	4	3	41	4	11	4	5	8	1	0	0	0	2	0	0	0	0	0	0	0	31	73	104	1.24	0.31	
Alpha-adducin OS=Mus musculus GN=Add1 PE=1 SV=2	Q9QYC0	2	3	3	4	0	0	33	0	7	0	0	0	2	4	6	6	0	0	2	6	0	0	0	0	30	48	78	0.51	0.00	
DNA-binding protein RFX6 OS=Mus musculus GN=Rfx6 PE=1 SV=2	Q8C7R7	7	8	5	5	2	1	1	5	3	5	4	6	0	0	0	0	0	0	0	0	0	0	0	0	28	24	52	0.41	1.68	
Disks large-associated protein 1 OS=Mus musculus GN=Dlgap1 PE=1 SV=3	Q9D415	0	0	0	0	0	0	0	0	0	0	0	0	4	7	5	5	3	1	9	15	3	3	3	1	25	34	59	0.57	0.26	
Myelin proteolipid protein OS=Mus musculus GN=Plp1 PE=1 SV=2	P60202	2	2	3	3	1	3	24	5	10	4	2	1	2	2	1	1	2	2	2	2	0	0	1	0	24	51	75	1.02	0.19	
Serine/threonine-protein phosphatase PP1-gamma catalytic subunit OS=Mus musculus GN=Ppp1cc PE=1 SV=1	P63087	2	2	2	2	2	2	2	3	3	2	4	3	1	2	2	2	2	1	2	2	2	4	2	3	22	32	54	1.02	1.29	
Actin, alpha skeletal muscle OS=Mus musculus GN=Acta1 PE=1 SV=1	P68134	3	5	3	4	3	3	10	5	5	6	12	6	0	0	0	0	0	0	0	0	0	0	0	0	21	44	65	0.93	1.21	
Synaptotagmin-7 OS=Mus musculus GN=Syt7 PE=1 SV=1	Q9R0N7	2	3	2	2	2	2	5	5	7	3	5	3	1	1	2	2	2	1	1	1	0	0	3	2	21	35	56	0.93	0.85	
Glial fibrillary acidic protein OS=Mus musculus GN=Gfap PE=1 SV=4	P03995	0	0	0	0	0	0	3	1	6	3	0	6	2	1	3	5	7	1	14	24	7	59	22	17	19	162	181	2.21	1.96	
RNA-binding motif protein, X chromosome OS=Mus musculus GN=Rbmx PE=1 SV=1	Q9WV02	3	6	1	4	2	3	4	4	12	5	8	3	0	0	0	0	0	0	0	0	0	0	0	0	19	36	55	0.92	0.80	
Unconventional myosin-Id OS=Mus musculus GN=Myo1d PE=1 SV=1	Q5SYD0	1	5	7	2	0	0	4	2	6	7	9	3	1	0	0	0	0	0	1	5	1	25	3	2	16	68	84	0.15	2.59	
Dynamin-1 OS=Mus musculus GN=Dnm1 PE=1 SV=2	P39053	0	1	11	2	0	0	162	20	42	1	0	3	0	0	0	0	0	0	0	0	0	0	0	0	14	228	242	0.17	0.02	
Core histone macro-H2A.1 OS=Mus musculus GN=H2afy PE=1 SV=3	Q9QZQ8	1	1	1	2	2	0	5	1	3	3	3	4	0	0	1	3	0	0	6	8	4	4	8	4	11	53	64	1.79	0.97	
Plectin OS=Mus musculus GN=Plec PE=1 SV=2	Q9QXS1	1	1	1	1	3	1	6	1	7	2	3	1	0	1	1	0	0	0	6	43	4	6	23	15	10	117	127	1.02	0.75	
Syntaxin-binding protein 1 OS=Mus musculus GN=Stxbp1 PE=1 SV=1	O08599	3	0	2	1	0	0	34	3	13	1	0	0	0	0	0	0	0	0	0	0	0	0	0	0	6	51	57	0.20	0.02	
Sodium/potassium-transporting ATPase subunit alpha-1 OS=Mus musculus GN=Atp1a1 PE=1 SV=1	Q8VDN2	1	0	1	0	3	0	92	3	9	0	0	0	0	0	0	0	0	0	0	0	0	0	0	0	5	104	109	1.53	0.00	
Plasma membrane calcium-transporting ATPase 2 OS=Mus musculus GN=Atp2b2 PE=1 SV=2	Q9R0K7	0	0	2	3	0	0	52	0	1	3	2	0	0	0	0	0	0	0	0	0	0	0	0	0	5	58	63	1.53	0.09	
Amphiphysin OS=Mus musculus GN=Amph PE=1 SV=1	Q7TQF7	2	1	0	1	0	0	44	1	0	1	1	2	0	0	0	0	0	0	0	0	0	0	0	0	4	49	53	0.34	0.09	
Band 4.1-like protein 3 OS=Mus musculus GN=Epb41l3 PE=1 SV=1	Q9WV92	0	0	0	0	0	0	42	0	2	0	0	0	0	0	0	2	0	1	4	10	1	2	5	0	3	66	69	#DIV/0!	0.12	
Aconitate hydratase, mitochondrial OS=Mus musculus GN=Aco2 PE=1 SV=1	Q99KI0	3	0	0	0	0	0	36	3	12	0	0	0	0	0	0	0	0	0	0	0	0	0	0	0	3	51	54	0.00	0.00	
Synapsin-1 OS=Mus musculus GN=Syn1 PE=1 SV=2	O88935	0	1	0	1	0	0	40	3	13	0	0	0	0	0	0	0	0	0	0	0	0	0	0	0	2	56	58	1.02	0.00	
Nidogen-2 OS=Mus musculus GN=Nid2 PE=1 SV=2	O88322	0	0	1	0	0	0	6	2	2	23	23	15	0	0	0	0	0	0	20	31	13	5	18	18	1	176	177	0.00	1.39	
Nidogen-1 OS=Mus musculus GN=Nid1 PE=1 SV=2	P10493	0	0	0	0	0	0	5	5	4	21	12	18	0	0	1	0	0	0	5	9	18	0	15	15	1	127	128	0.00	1.77	
Laminin subunit beta-2 OS=Mus musculus GN=Lamb2 PE=2 SV=2	Q61292	0	0	0	0	0	0	18	18	17	26	33	22	0	0	0	0	0	0	15	15	15	10	21	22	0	232	232	#DIV/0!	1.37	
Sodium/potassium-transporting ATPase subunit alpha-2 OS=Mus musculus GN=Atp1a2 PE=1 SV=1	Q6PIE5	0	0	0	0	0	0	172	11	24	0	0	0	0	0	0	0	0	0	0	0	0	0	0	0	0	207	207	#DIV/0!	0.00	
Laminin subunit gamma-1 OS=Mus musculus GN=Lamc1 PE=1 SV=2	P02468	0	0	0	0	0	0	11	8	8	21	23	23	0	0	0	0	0	0	11	12	9	8	19	24	0	177	177	#DIV/0!	2.01	
Ubiquitin-like modifier-activating enzyme 1 OS=Mus musculus GN=Uba1 PE=1 SV=1	Q02053	0	0	0	0	0	0	158	4	14	0	0	0	0	0	0	0	0	0	0	0	0	0	0	0	0	176	176	#DIV/0!	0.00	
Hexokinase-1 OS=Mus musculus GN=Hk1 PE=1 SV=3	P17710	0	0	0	0	0	0	111	5	5	0	0	0	0	0	0	0	0	0	0	0	0	0	0	0	0	121	121	#DIV/0!	0.00	
Sarcoplasmic/endoplasmic reticulum calcium ATPase 2 OS=Mus musculus GN=Atp2a2 PE=1 SV=2	O55143	0	0	0	0	0	0	77	11	3	0	0	0	0	0	0	0	0	0	0	0	0	0	0	0	0	91	91	#DIV/0!	0.00	
Basement membrane-specific heparan sulfate proteoglycan core protein OS=Mus musculus GN=Hspg2 PE=1 SV=1	Q05793	0	0	0	0	0	0	7	4	5	12	16	13	0	0	0	0	0	0	1	3	5	0	9	7	0	82	82	#DIV/0!	2.29	
Myosin-11 OS=Mus musculus GN=Myh11 PE=1 SV=1	O08638	0	0	0	0	0	0	0	5	1	6	1	4	0	0	0	0	0	0	9	3	1	1	13	14	0	58	58	#DIV/0!	2.06	
Endoplasmin OS=Mus musculus GN=Hsp90b1 PE=1 SV=2	P08113	0	0	0	0	0	0	46	0	3	0	1	0	0	0	0	0	0	0	0	0	0	0	0	0	0	50	50	#DIV/0!	0.02	

Name	Accession Number	S2 Spectral Counts	S3 Spectral Counts	Total Spectral Counts	S2 Lesion/Intact Normalized Ratio	S3 Lesion/Intact Normalized Ratio
SRC kinase signaling inhibitor 1 OS=Mus musculus GN=Srcin1 PE=1 SV=2	sp|Q9QWI6|SRCN1_MOUSE	4030	630	4660	1.10	0.85
Neurabin-2 OS=Mus musculus GN=Ppp1r9b PE=1 SV=1	sp|Q6R891|NEB2_MOUSE	1110	2872	3982	1.00	1.00
Neurofilament medium polypeptide OS=Mus musculus GN=Nefm PE=1 SV=4	sp|P08553|NFM_MOUSE	960	1874	2834	0.92	0.82
Ras GTPase-activating protein SynGAP OS=Mus musculus GN=Syngap1 PE=3 SV=2	sp|F6SEU4|SYGP1_MOUSE	1146	1195	2341	0.82	0.91
ERC protein 2 OS=Mus musculus GN=Erc2 PE=1 SV=2	sp|Q6PH08|ERC2_MOUSE	775	825	1600	1.05	0.93
Clathrin heavy chain 1 OS=Mus musculus GN=Cltc PE=1 SV=3	sp|Q68FD5|CLH_MOUSE	1365	172	1537	1.66	0.15
Breakpoint cluster region protein OS=Mus musculus GN=Bcr PE=1 SV=3	sp|Q6PAJ1|BCR_MOUSE	1111	1	1112	0.99	0.00
Spectrin alpha chain, brain OS=Mus musculus GN=Sptan1 PE=1 SV=4	sp|P16546|SPTA2_MOUSE	593	491	1084	0.98	0.58
Disks large homolog 4 OS=Mus musculus GN=Dlg4 PE=1 SV=1	sp|Q62108|DLG4_MOUSE	431	649	1080	1.35	1.07
Sodium/potassium-transporting ATPase subunit alpha-3 OS=Mus musculus GN=Atp1a3 PE=1 SV=1	sp|Q6PIC6|AT1A3_MOUSE	224	834	1058	0.92	0.03
Actin, cytoplasmic 1 OS=Mus musculus GN=Actb PE=1 SV=1	sp|P60710|ACTB_MOUSE (+1)	501	427	928	0.99	1.25
Myosin-10 OS=Mus musculus GN=Myh10 PE=1 SV=2	sp|Q61879|MYH10_MOUSE	670	215	885	0.99	0.98
Drebrin OS=Mus musculus GN=Dbn1 PE=1 SV=4	sp|Q9QXS6|DREB_MOUSE	642	218	860	0.98	0.49
Protein phosphatase 1 regulatory subunit 12A OS=Mus musculus GN=Ppp1r12a PE=1 SV=2	sp|Q9DBR7|MYPT1_MOUSE	816	34	850	1.11	0.22
Glutamate [NMDA] receptor subunit zeta-1 OS=Mus musculus GN=Grin1 PE=1 SV=1	sp|P35438|NMDZ1_MOUSE	433	268	701	0.76	0.72
Desmoplakin OS=Mus musculus GN=Dsp PE=3 SV=1	sp|E9Q557|DESP_MOUSE	430	258	688	0.86	1.12
Heterogeneous nuclear ribonucleoprotein U OS=Mus musculus GN=Hnrnpu PE=1 SV=1	sp|Q8VEK3|HNRPU_MOUSE	244	440	684	1.02	0.86
Glycogen phosphorylase, muscle form OS=Mus musculus GN=Pygm PE=1 SV=3	sp|Q9WUB3|PYGM_MOUSE	313	302	615	1.33	1.01
Unconventional myosin-Va OS=Mus musculus GN=Myo5a PE=1 SV=2	sp|Q99104|MYO5A_MOUSE	393	169	562	0.87	0.93
Alpha-actinin-1 OS=Mus musculus GN=Actn1 PE=1 SV=1	sp|Q7TPR4|ACTN1_MOUSE	437	111	548	1.08	0.29
Disks large homolog 2 OS=Mus musculus GN=Dlg2 PE=1 SV=1	sp|Q91XM9|DLG2_MOUSE	153	354	507	0.91	1.17
Neurofilament light polypeptide OS=Mus musculus GN=Nefl PE=1 SV=5	sp|P08551|NFL_MOUSE	166	333	499	0.86	1.11
IQ motif and SEC7 domain-containing protein 1 OS=Mus musculus GN=Iqsec1 PE=1 SV=2	sp|Q8R0S2|IQEC1_MOUSE	338	149	487	1.18	0.64
Tubulin alpha-1A chain OS=Mus musculus GN=Tuba1a PE=1 SV=1	sp|P68369|TBA1A_MOUSE (+1)	315	162	477	1.17	0.73
Alpha-internexin OS=Mus musculus GN=Ina PE=1 SV=2	sp|P46660|AINX_MOUSE	155	318	473	1.21	1.22
Contactin-1 OS=Mus musculus GN=Cntn1 PE=1 SV=1	sp|P12960|CNTN1_MOUSE	339	132	471	1.30	0.17
Protein bassoon OS=Mus musculus GN=Bsn PE=1 SV=4	sp|O88737|BSN_MOUSE	452	12	464	1.18	0.72
Spectrin beta chain, brain 1 OS=Mus musculus GN=Sptbn1 PE=1 SV=2	sp|Q62261|SPTB2_MOUSE	256	197	453	0.95	0.81
Serine/threonine-protein phosphatase PP1-alpha catalytic subunit OS=Mus musculus GN=Ppp1ca PE=1 SV=1	sp|P62137|PP1A_MOUSE	139	296	435	0.92	1.28
Neural cell adhesion molecule 1 OS=Mus musculus GN=Ncam1 PE=1 SV=3	sp|P13595|NCAM1_MOUSE	246	170	416	0.94	0.46
Disks large-associated protein 3 OS=Mus musculus GN=Dlgap3 PE=1 SV=1	sp|Q6PFD5|DLGP3_MOUSE	131	269	400	0.43	0.80
Junction plakoglobin OS=Mus musculus GN=Jup PE=1 SV=3	sp|Q02257|PLAK_MOUSE	282	118	400	0.67	0.85
Myosin-9 OS=Mus musculus GN=Myh9 PE=1 SV=4	sp|Q8VDD5|MYH9_MOUSE	149	239	388	0.96	1.24
V-type proton ATPase 116 kDa subunit a isoform 1 OS=Mus musculus GN=Atp6v0a1 PE=1 SV=3	sp|Q9Z1G4|VPP1_MOUSE	232	126	358	0.82	0.02
ATP-dependent RNA helicase A OS=Mus musculus GN=Dhx9 PE=1 SV=2	sp|O70133|DHX9_MOUSE	299	58	357	1.10	1.01
AP-2 complex subunit alpha-1 OS=Mus musculus GN=Ap2a1 PE=1 SV=1	sp|P17426|AP2A1_MOUSE	199	157	356	0.47	0.05
Arf-GAP with GTPase, ANK repeat and PH domain-containing protein 2 OS=Mus musculus GN=Agap2 PE=1 SV=1	sp|Q3UHD9|AGAP2_MOUSE	270	67	337	1.36	0.32
AP-2 complex subunit beta OS=Mus musculus GN=Ap2b1 PE=1 SV=1	sp|Q9DBG3|AP2B1_MOUSE	130	202	332	1.16	0.06
SH3 and multiple ankyrin repeat domains protein 3 OS=Mus musculus GN=Shank3 PE=1 SV=2	sp|Q4ACU6|SHAN3_MOUSE	153	176	329	0.84	0.75
Matrin-3 OS=Mus musculus GN=Matr3 PE=1 SV=1	sp|Q8K310|MATR3_MOUSE	260	46	306	1.23	0.84
2',3'-cyclic-nucleotide 3'-phosphodiesterase OS=Mus musculus GN=Cnp PE=1 SV=3	sp|P16330|CN37_MOUSE	147	158	305	0.86	0.55
Histone H1.3 OS=Mus musculus GN=Hist1h1d PE=1 SV=2	sp|P43277|H13_MOUSE	61	243	304	1.13	1.30
Myosin-4 OS=Mus musculus GN=Myh4 PE=1 SV=1	sp|Q5SX39|MYH4_MOUSE	90	213	303	1.34	1.03
LisH domain and HEAT repeat-containing protein KIAA1468 OS=Mus musculus GN=Kiaa1468 PE=1 SV=1	sp|Q148V7|K1468_MOUSE	291	7	298	1.38	6.03
Unconventional myosin-VI OS=Mus musculus GN=Myo6 PE=1 SV=1	sp|Q64331|MYO6_MOUSE	270	22	292	1.05	0.38
Gap junction alpha-1 protein OS=Mus musculus GN=Gja1 PE=1 SV=2	sp|P23242|CXA1_MOUSE	128	157	285	0.99	1.10
Heat shock cognate 71 kDa protein OS=Mus musculus GN=Hspa8 PE=1 SV=1	sp|P63017|HSP7C_MOUSE	181	95	276	0.88	0.40
Alpha-actinin-2 OS=Mus musculus GN=Actn2 PE=1 SV=1	sp|Q9JI91|ACTN2_MOUSE	249	23	272	0.77	0.77
Myelin basic protein OS=Mus musculus GN=Mbp PE=1 SV=2	sp|P04370|MBP_MOUSE	187	85	272	1.15	1.13
Dynamin-1 OS=Mus musculus GN=Dnm1 PE=1 SV=2	sp|P39053|DYN1_MOUSE	14	228	242	0.17	0.02
Laminin subunit beta-2 OS=Mus musculus GN=Lamb2 PE=2 SV=2	sp|Q61292|LAMB2_MOUSE	0	232	232	#DIV/0!	1.37

Disks large-associated protein 2 OS=Mus musculus GN=Dlgap2 PE=1 SV=2	sp|Q8BJ42|DLGP2_MOUSE	99	126	225	0.69	0.47
Calcium/calmodulin-dependent protein kinase type II subunit beta OS=Mus musculus GN=Camk2b PE=1 SV=2	sp|P28652|KCC2B_MOUSE	113	110	223	0.78	0.57
Calcium/calmodulin-dependent protein kinase type II subunit alpha OS=Mus musculus GN=Camk2a PE=1 SV=2	sp|P11798|KCC2A_MOUSE	80	142	222	0.79	1.01
Dihydropyrimidinase-related protein 2 OS=Mus musculus GN=Dpysl2 PE=1 SV=2	sp|O08553|DPYL2_MOUSE	141	80	221	1.01	0.36
Rap guanine nucleotide exchange factor 4 OS=Mus musculus GN=Rapgef4 PE=1 SV=1	sp|Q9EQZ6|RPGF4_MOUSE	195	21	216	1.03	1.34
Sodium/potassium-transporting ATPase subunit alpha-2 OS=Mus musculus GN=Atp1a2 PE=1 SV=1	sp|Q6PIE5|AT1A2_MOUSE	0	207	207	#DIV/0!	0.00
Catenin alpha-2 OS=Mus musculus GN=Ctnna2 PE=1 SV=3	sp|Q61301|CTNA2_MOUSE	161	44	205	1.20	0.05
Microtubule-associated protein 6 OS=Mus musculus GN=Map6 PE=1 SV=2	sp|Q7TSJ2|MAP6_MOUSE	122	61	183	1.42	0.02
Synaptopodin OS=Mus musculus GN=Synpo PE=1 SV=2	sp|Q8CC35|SYNPO_MOUSE	175	8	183	0.67	0.34
Glial fibrillary acidic protein OS=Mus musculus GN=Gfap PE=1 SV=4	sp|P03995|GFAP_MOUSE	19	162	181	2.21	1.96
F-box only protein 41 OS=Mus musculus GN=Fbxo41 PE=1 SV=3	sp|Q6NS60|FBX41_MOUSE	89	89	178	0.80	2.31
Laminin subunit gamma-1 OS=Mus musculus GN=Lamc1 PE=1 SV=2	sp|P02468|LAMC1_MOUSE	0	177	177	#DIV/0!	2.01
Nidogen-2 OS=Mus musculus GN=Nid2 PE=1 SV=2	sp|O88322|NID2_MOUSE	1	176	177	0.00	1.39
Ubiquitin-like modifier-activating enzyme 1 OS=Mus musculus GN=Uba1 PE=1 SV=1	sp|Q02053|UBA1_MOUSE	0	176	176	#DIV/0!	0.00
A-kinase anchor protein 5 OS=Mus musculus GN=Akap5 PE=3 SV=2	sp|D3YVF0|AKAP5_MOUSE	129	45	174	0.89	3.11
Unconventional myosin-Ib OS=Mus musculus GN=Myo1b PE=2 SV=3	sp|P46735|MYO1B_MOUSE	59	104	163	0.75	0.80
Guanine nucleotide-binding protein G(o) subunit alpha OS=Mus musculus GN=Gnao1 PE=1 SV=3	sp|P18872|GNAO_MOUSE	94	68	162	0.90	0.51
Protein piccolo OS=Mus musculus GN=Pclo PE=1 SV=3	sp|Q9QYX7|PCLO_MOUSE	134	23	157	1.42	1.10
Calcium-binding mitochondrial carrier protein Aralar1 OS=Mus musculus GN=Slc25a12 PE=1 SV=1	sp|Q8BH59|CMC1_MOUSE	131	20	151	0.95	0.00
Tubulin alpha-1B chain OS=Mus musculus GN=Tuba1b PE=1 SV=2	sp|P05213|TBA1B_MOUSE	50	97	147	0.80	0.74
cAMP and cAMP-inhibited cGMP 3',5'-cyclic phosphodiesterase 10A OS=Mus musculus GN=Pde10a PE=1 SV=2	sp|Q8CA95|PDE10_MOUSE	137	2	139	0.90	0.00
Copine-5 OS=Mus musculus GN=Cpne5 PE=2 SV=1	sp|Q8JZW4|CPNE5_MOUSE	76	58	134	0.87	0.53
Tubulin beta-5 chain OS=Mus musculus GN=Tubb5 PE=1 SV=1	sp|P99024|TBB5_MOUSE	77	57	134	1.23	0.33
Homer protein homolog 1 OS=Mus musculus GN=Homer1 PE=1 SV=2	sp|Q9Z2Y3|HOME1_MOUSE	45	86	131	0.56	1.01
Nidogen-1 OS=Mus musculus GN=Nid1 PE=1 SV=2	sp|P10493|NID1_MOUSE	1	127	128	0.00	1.77
Plectin OS=Mus musculus GN=Plec PE=1 SV=2	sp|Q9QXS1|PLEC_MOUSE	10	117	127	1.02	0.75
Hexokinase-1 OS=Mus musculus GN=Hk1 PE=1 SV=3	sp|P17710|HXK1_MOUSE	0	121	121	#DIV/0!	0.00
Sickle tail protein OS=Mus musculus GN=Skt PE=1 SV=1	sp|A2AQ25|SKT_MOUSE	117	4	121	1.04	0.00
Mitogen-activated protein kinase kinase kinase 5 OS=Mus musculus GN=Map3k5 PE=1 SV=3	sp|O35099|M3K5_MOUSE	114	0	114	1.14	#DIV/0!
Tubulin beta-4B chain OS=Mus musculus GN=Tubb4b PE=1 SV=1	sp|P68372|TBB4B_MOUSE	69	43	112	1.05	0.60
Sodium/potassium-transporting ATPase subunit alpha-1 OS=Mus musculus GN=Atp1a1 PE=1 SV=1	sp|Q8VDN2|AT1A1_MOUSE	5	104	109	1.53	0.00
Brain-specific angiogenesis inhibitor 1-associated protein 2 OS=Mus musculus GN=Baiap2 PE=1 SV=2	sp|Q8BKX1|BAIP2_MOUSE	83	23	106	1.15	0.54
Glyceraldehyde-3-phosphate dehydrogenase OS=Mus musculus GN=Gapdh PE=1 SV=2	sp|P16858|G3P_MOUSE	31	73	104	1.24	0.31
AP-2 complex subunit alpha-2 OS=Mus musculus GN=Ap2a2 PE=1 SV=2	sp|P17427|AP2A2_MOUSE	43	60	103	0.35	0.00
Glycogen phosphorylase, muscle form OS=Mus musculus GN=Pygm PE=1 SV=3	sp|Q9WUB3|PYGM_MOUSE	50	52	102	0.87	0.58
Valine--tRNA ligase OS=Mus musculus GN=Vars PE=2 SV=1	sp|Q9Z1Q9|SYVC_MOUSE	98	0	98	2.83	#DIV/0!
Annexin A2 OS=Mus musculus GN=Anxa2 PE=1 SV=2	sp|P07356|ANXA2_MOUSE	50	42	92	0.63	1.01
Sarcoplasmic/endoplasmic reticulum calcium ATPase 2 OS=Mus musculus GN=Atp2a2 PE=1 SV=2	sp|O55143|AT2A2_MOUSE	0	91	91	#DIV/0!	0.00
Unconventional myosin-Id OS=Mus musculus GN=Myo1d PE=1 SV=1	sp|Q5SYD0|MYO1D_MOUSE	16	68	84	0.15	2.59
Basement membrane-specific heparan sulfate proteoglycan core protein OS=Mus musculus GN=Hspg2 PE=1 SV=1	sp|Q05793|PGBM_MOUSE	0	82	82	#DIV/0!	2.29
MTSS1-like protein OS=Mus musculus GN=Mtss1l PE=1 SV=1	sp|Q6P9S0|MTSSL_MOUSE	79	0	79	0.86	#DIV/0!
Alpha-adducin OS=Mus musculus GN=Add1 PE=1 SV=2	sp|Q9QYC0|ADDA_MOUSE	30	48	78	0.51	0.00
Transcriptional activator protein Pur-alpha OS=Mus musculus GN=Pura PE=1 SV=1	sp|P42669|PURA_MOUSE	72	6	78	1.08	0.00
Myelin proteolipid protein OS=Mus musculus GN=Plp1 PE=1 SV=2	sp|P60202|MYPR_MOUSE	24	51	75	1.02	0.19
ATP synthase subunit gamma, mitochondrial OS=Mus musculus GN=Atp5c1 PE=1 SV=1	sp|Q91VR2|ATPG_MOUSE	61	11	72	0.93	0.00
Regulator of nonsense transcripts 1 OS=Mus musculus GN=Upf1 PE=1 SV=2	sp|Q9EPU0|RENT1_MOUSE	71	0	71	0.84	#DIV/0!
Band 4.1-like protein 3 OS=Mus musculus GN=Epb41l3 PE=1 SV=1	sp|Q9WV92|E41L3_MOUSE	3	66	69	#DIV/0!	0.12
Oligodendrocyte-myelin glycoprotein OS=Mus musculus GN=Omg PE=1 SV=1	sp|Q63912|OMGP_MOUSE	62	7	69	1.16	0.00
Protein Dok-7 OS=Mus musculus GN=Dok7 PE=1 SV=1	sp|Q18PE0|DOK7_MOUSE	36	33	69	0.82	0.95
Actin, alpha skeletal muscle OS=Mus musculus GN=Acta1 PE=1 SV=1	sp|P68134|ACTS_MOUSE	21	44	65	0.93	1.21
Centromere protein V OS=Mus musculus GN=Cenpv PE=2 SV=2	sp|Q9CXS4|CENPV_MOUSE	37	28	65	0.87	0.65
Core histone macro-H2A.1 OS=Mus musculus GN=H2afy PE=1 SV=3	sp|Q9QZQ8|H2AY_MOUSE	11	53	64	1.79	0.97
Guanine nucleotide-binding protein G(z) subunit alpha OS=Mus musculus GN=Gnaz PE=2 SV=4	sp|O70443|GNAZ_MOUSE	60	3	63	0.84	0.00

Plasma membrane calcium-transporting ATPase 2 OS=Mus musculus GN=Atp2b2 PE=1 SV=2	sp|Q9R0K7|AT2B2_MOUSE	5	58	63	1.53	0.09
60S acidic ribosomal protein P0 OS=Mus musculus GN=Rplp0 PE=1 SV=3	sp|P14869|RLA0_MOUSE	56	6	62	1.27	0.50
Calcium-activated potassium channel subunit alpha-1 OS=Mus musculus GN=Kcnma1 PE=1 SV=2	sp|Q08460|KCMA1_MOUSE	53	9	62	2.16	0.00
Guanine nucleotide-binding protein G(I)/G(S)/G(T) subunit beta-1 OS=Mus musculus GN=Gnb1 PE=1 SV=3	sp|P62874|GBB1_MOUSE	49	13	62	1.06	0.08
Disks large-associated protein 1 OS=Mus musculus GN=Dlgap1 PE=1 SV=3	sp|Q9D415|DLGP1_MOUSE	25	34	59	0.57	0.26
SRSF protein kinase 2 OS=Mus musculus GN=Srpk2 PE=1 SV=2	sp|O54781|SRPK2_MOUSE	59	0	59	2.15	#DIV/0!
Myosin-11 OS=Mus musculus GN=Myh11 PE=1 SV=1	sp|O08638|MYH11_MOUSE	0	58	58	#DIV/0!	2.06
Synapsin-1 OS=Mus musculus GN=Syn1 PE=1 SV=2	sp|O88935|SYN1_MOUSE	2	56	58	1.02	0.00
Tubulin beta-2A chain OS=Mus musculus GN=Tubb2a PE=1 SV=1	sp|Q7TMM9|TBB2A_MOUSE	36	22	58	1.28	0.38
ATPase family AAA domain-containing protein 3 OS=Mus musculus GN=Atad3 PE=1 SV=1	sp|Q925I1|ATAD3_MOUSE	57	0	57	1.14	#DIV/0!
Syntaxin-binding protein 1 OS=Mus musculus GN=Stxbp1 PE=1 SV=2	sp|O08599|STXB1_MOUSE	6	51	57	0.20	0.02
Desmoglein-1-alpha OS=Mus musculus GN=Dsg1a PE=2 SV=2	sp|Q61495|DSG1A_MOUSE (+1)	40	16	56	0.76	1.29
Synaptotagmin-7 OS=Mus musculus GN=Syt7 PE=1 SV=1	sp|Q9R0N7|SYT7_MOUSE	21	35	56	0.93	0.85
RNA-binding motif protein, X chromosome OS=Mus musculus GN=Rbmx PE=1 SV=1	sp|Q9WV02|RBMX_MOUSE	19	36	55	0.92	0.80
Aconitate hydratase, mitochondrial OS=Mus musculus GN=Aco2 PE=1 SV=1	sp|Q99KI0|ACON_MOUSE	3	51	54	0.00	0.00
Kinesin-like protein KIF2A OS=Mus musculus GN=Kif2a PE=1 SV=2	sp|P28740|KIF2A_MOUSE	35	19	54	0.35	0.12
Serine/threonine-protein phosphatase PP1-gamma catalytic subunit OS=Mus musculus GN=Ppp1cc PE=1 SV=1	sp|P63087|PP1G_MOUSE	22	32	54	1.02	1.29
Amphiphysin OS=Mus musculus GN=Amph PE=1 SV=1	sp|Q7TQF7|AMPH_MOUSE	4	49	53	0.34	0.09
DNA-binding protein RFX6 OS=Mus musculus GN=Rfx6 PE=1 SV=2	sp|Q8C7R7|RFX6_MOUSE	28	24	52	0.41	1.68
Endoplasmin OS=Mus musculus GN=Hsp90b1 PE=1 SV=2	sp|P08113|ENPL_MOUSE	0	50	50	#DIV/0!	0.02
Tubulin alpha-4A chain OS=Mus musculus GN=Tuba4a PE=1 SV=1	sp|P68368|TBA4A_MOUSE	32	17	49	1.31	0.89

Figure S1A

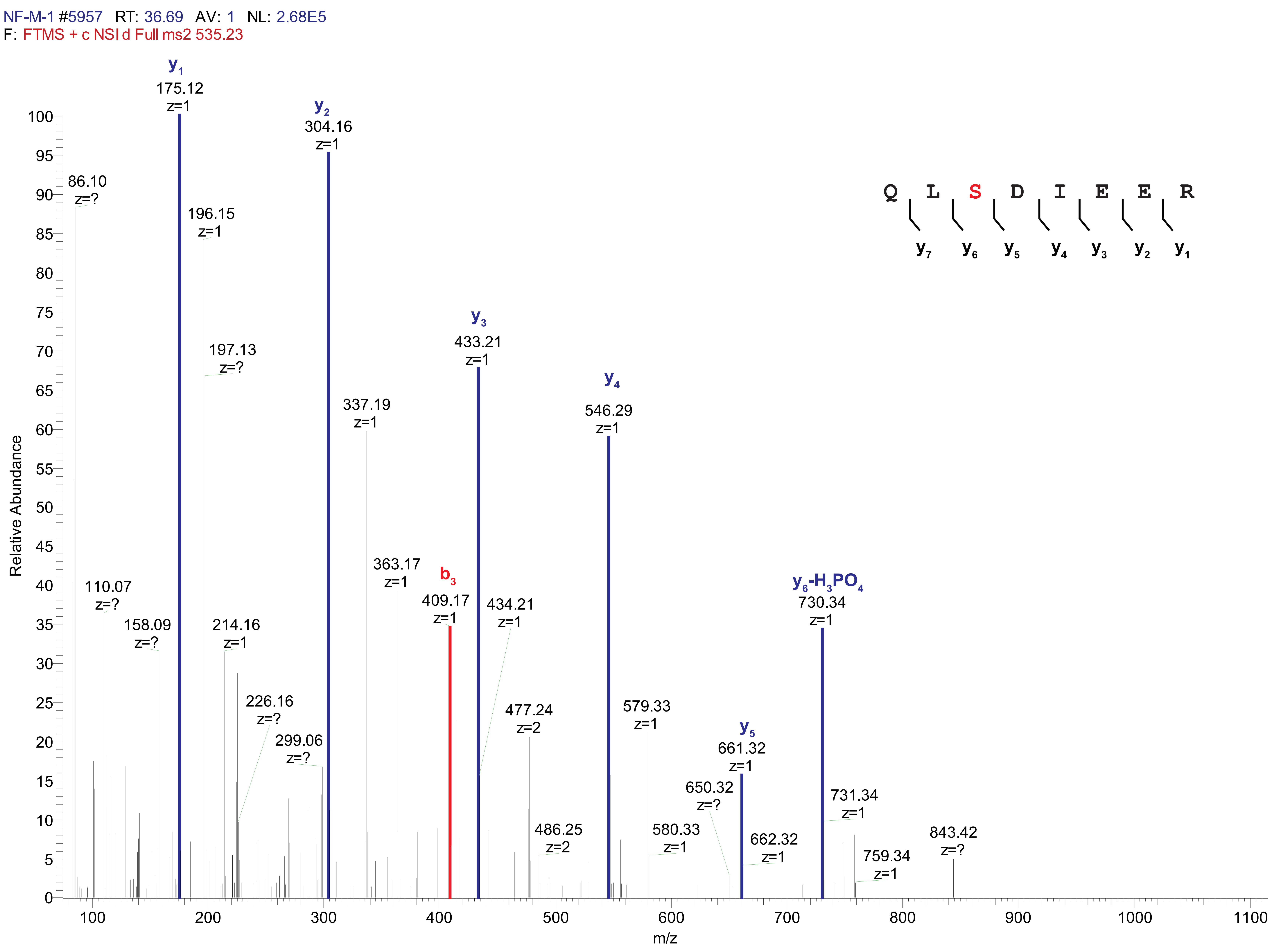

NF-M-1 #5957 RT: 36.69 AV: 1 NL: 2.68E5
F: FTMS + c NSI d Full ms2 535.23
Q L S D I E E R
y7 y6 y5 y4 y3 y2 y1
y1 175.12 z=1
y2 304.16 z=1
y3 433.21 z=1
y4 546.29 z=1
y5 661.32 z=1
b3 409.17 z=1
y6-H3PO4 730.34 z=1
86.10 z=?
196.15 z=1
197.13 z=?
337.19 z=1
363.17 z=1
434.21 z=1
477.24 z=2
579.33 z=1
110.07 z=?
158.09 z=?
214.16 z=1
226.16 z=?
299.06 z=?
486.25 z=2
580.33 z=1
650.32 z=?
662.32 z=1
731.34 z=1
759.34 z=1
843.42 z=?
Relative Abundance
m/z

Figure S1B

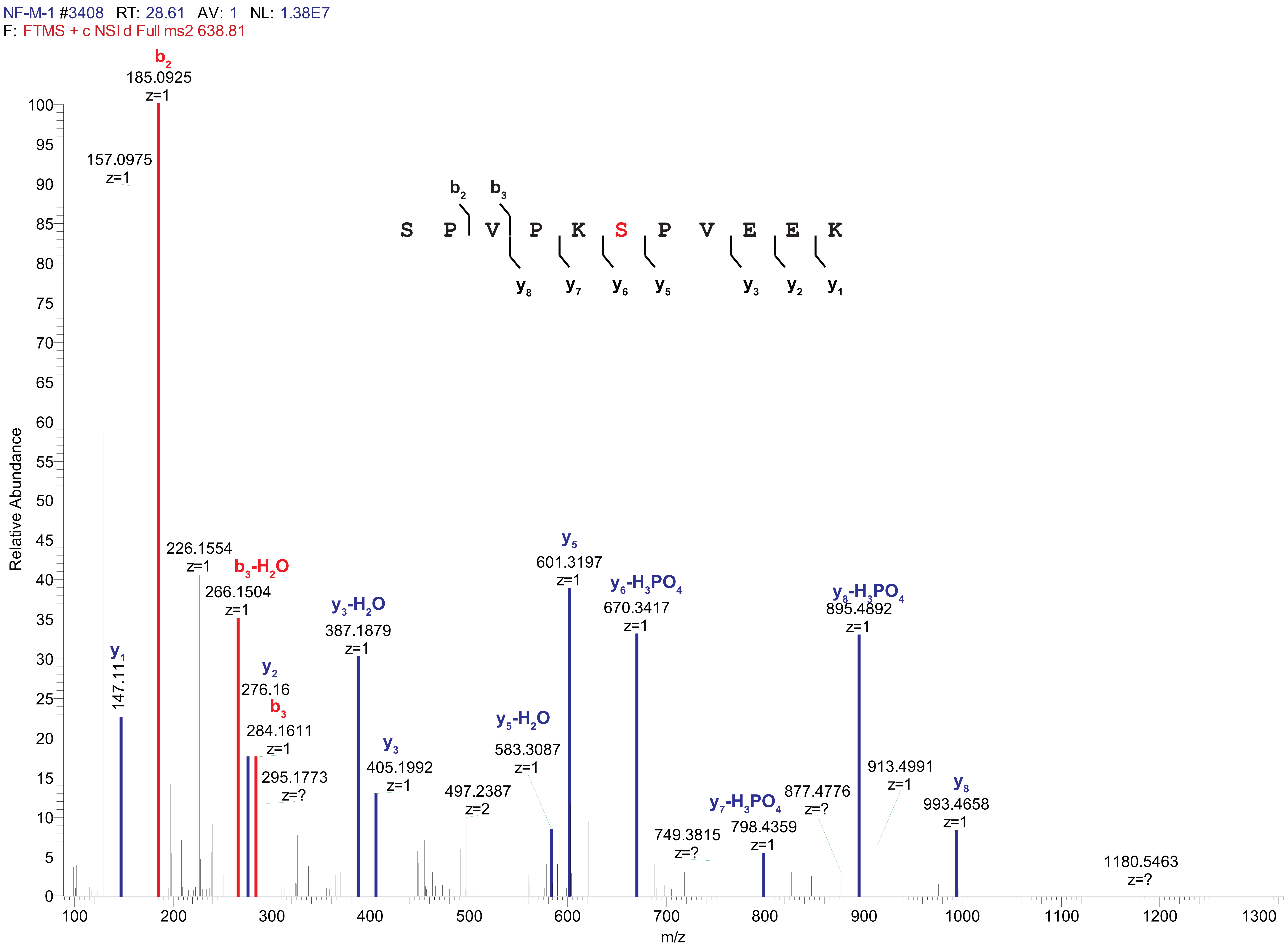

NF-M-1 #3408 RT: 28.61 AV: 1 NL: 1.38E7
F: FTMS + c NSI d Full ms2 638.81
S P V P K S P V E E K
b2 b3
y8 y7 y6 y5 y3 y2 y1
b2 185.0925 z=1
157.0975 z=1
226.1554 z=1
b3-H2O 266.1504 z=1
y1 147.11
y2 276.16
b3 284.1611 z=1
295.1773 z=?
y3-H2O 387.1879 z=1
y3 405.1992 z=1
497.2387 z=2
y5-H2O 583.3087 z=1
y5 601.3197 z=1
y6-H3PO4 670.3417 z=1
749.3815 z=?
y7-H3PO4 798.4359 z=1
877.4776 z=?
y8-H3PO4 895.4892 z=1
913.4991 z=1
y8 993.4658 z=1
1180.5463 z=?
Relative Abundance
m/z

Figure S1C

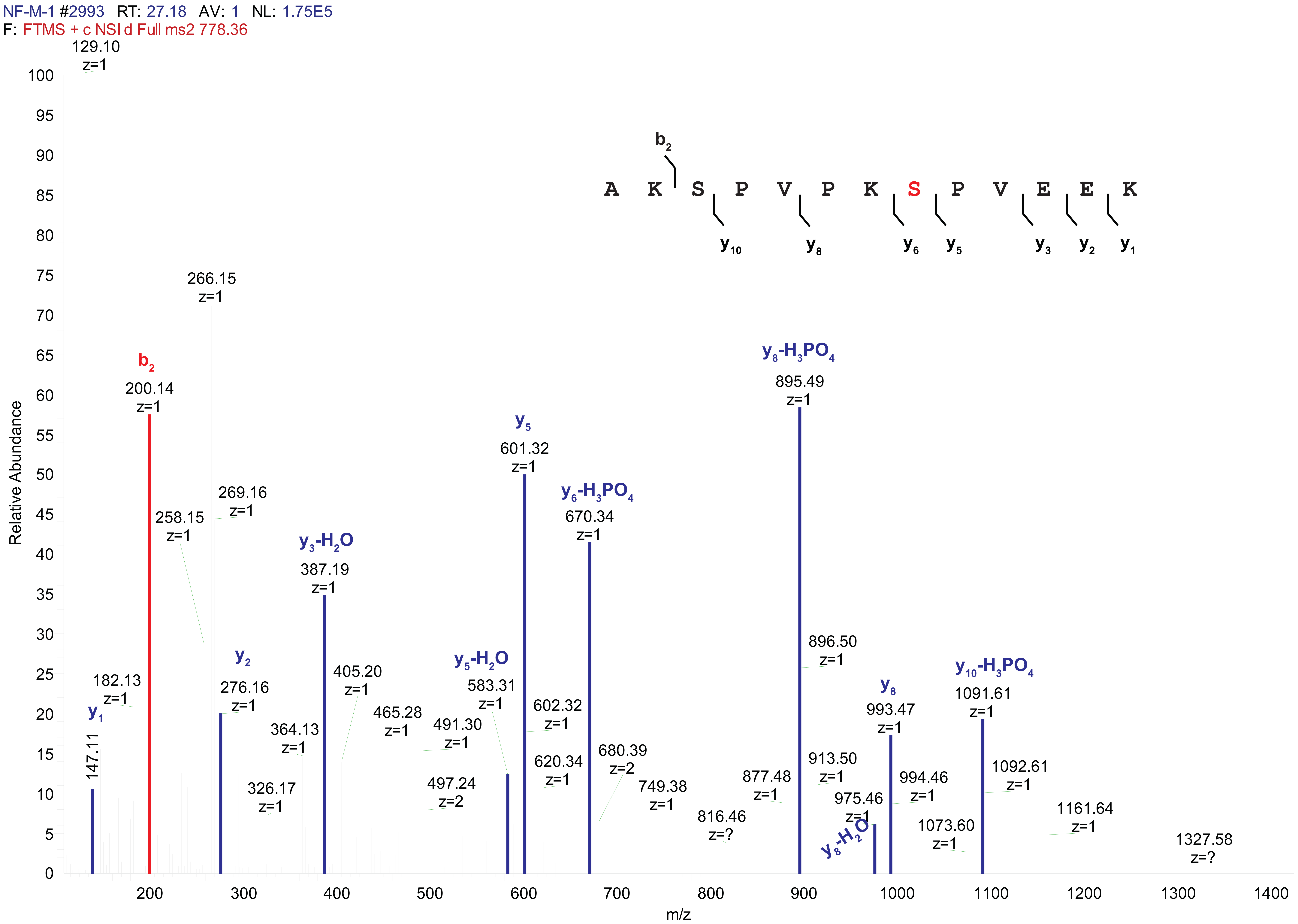
NF-M-1 #2993 RT: 27.18 AV: 1 NL: 1.75E5
F: FTMS + c NSI d Full ms2 778.36
Relative Abundance
m/z
A K S P V P K S P V E E K
b2
y10 y8 y6 y5 y3 y2 y1
129.10 z=1
y1 147.11
182.13 z=1
b2 200.14 z=1
258.15 z=1
266.15 z=1
269.16 z=1
y2 276.16 z=1
326.17 z=1
364.13 z=1
y3-H2O 387.19 z=1
405.20 z=1
465.28 z=1
491.30 z=1
497.24 z=2
y5-H2O 583.31 z=1
y5 601.32 z=1
602.32 z=1
620.34 z=1
y6-H3PO4 670.34 z=1
680.39 z=2
749.38 z=1
816.46 z=?
877.48 z=1
y8-H3PO4 895.49 z=1
896.50 z=1
913.50 z=1
975.46 z=1
y8-H2O
y8 993.47 z=1
994.46 z=1
1073.60 z=1
y10-H3PO4 1091.61 z=1
1092.61 z=1
1161.64 z=1
1327.58 z=?

Figure S1D

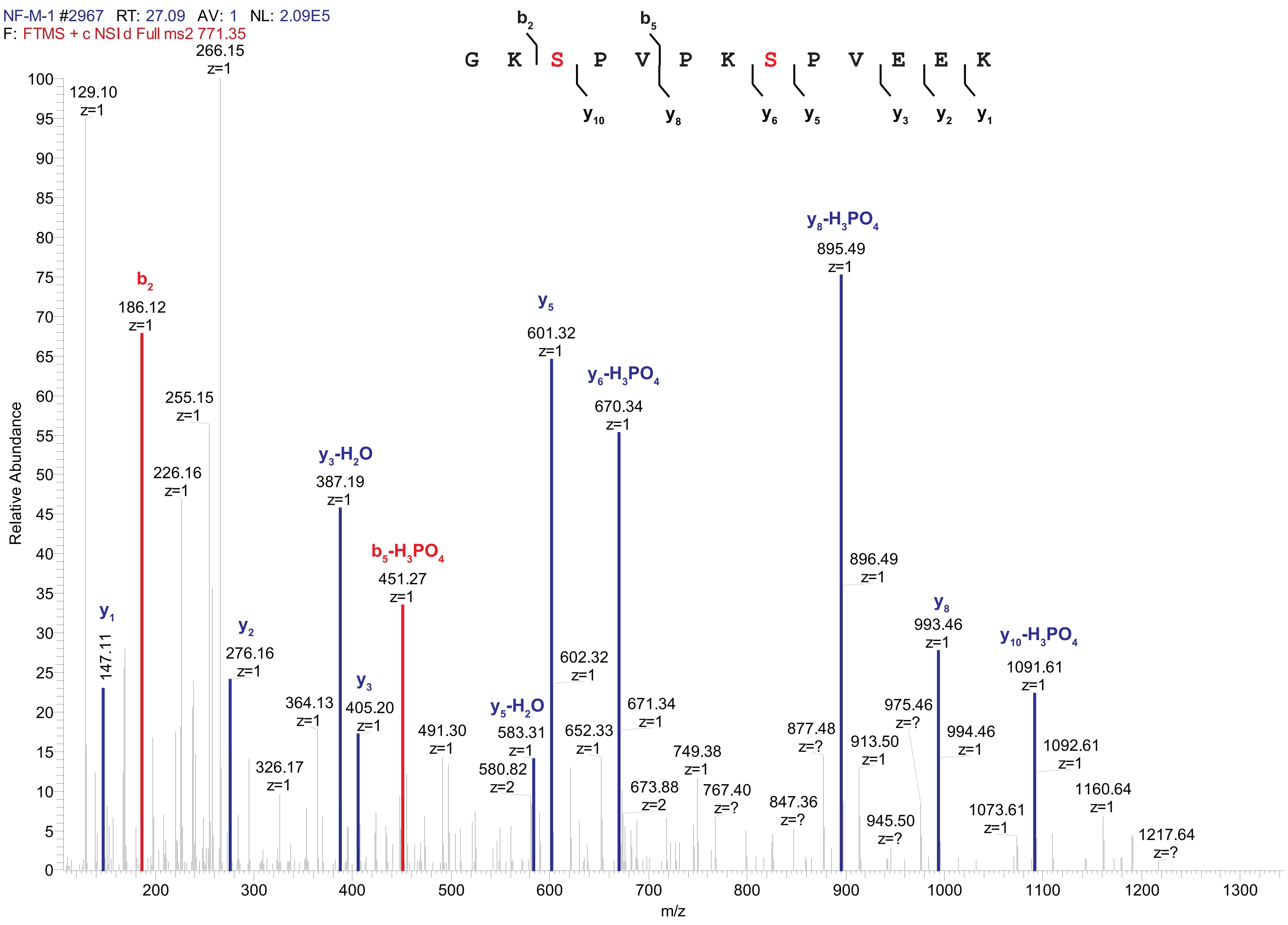

NF-M-1 #2967 RT: 27.09 AV: 1 NL: 2.09E5
F: FTMS + c NSI d Full ms2 771.35
G K S P V P K S P V E E K
b2
b5
y10
y8
y6
y5
y3
y2
y1
Relative Abundance
m/z
129.10 z=1
266.15 z=1
b2 186.12 z=1
255.15 z=1
226.16 z=1
y1 147.11
y2 276.16 z=1
326.17 z=1
364.13 z=1
y3-H2O 387.19 z=1
y3 405.20 z=1
b5-H3PO4 451.27 z=1
491.30 z=1
580.82 z=2
y5-H2O 583.31 z=1
y5 601.32 z=1
602.32 z=1
652.33 z=1
y6-H3PO4 670.34 z=1
671.34 z=1
673.88 z=2
749.38 z=1
767.40 z=?
847.36 z=?
877.48 z=?
y8-H3PO4 895.49 z=1
896.49 z=1
913.50 z=1
945.50 z=?
975.46 z=?
y8 993.46 z=1
994.46 z=1
1073.61 z=1
y10-H3PO4 1091.61 z=1
1092.61 z=1
1160.64 z=1
1217.64 z=?

Figure S1E

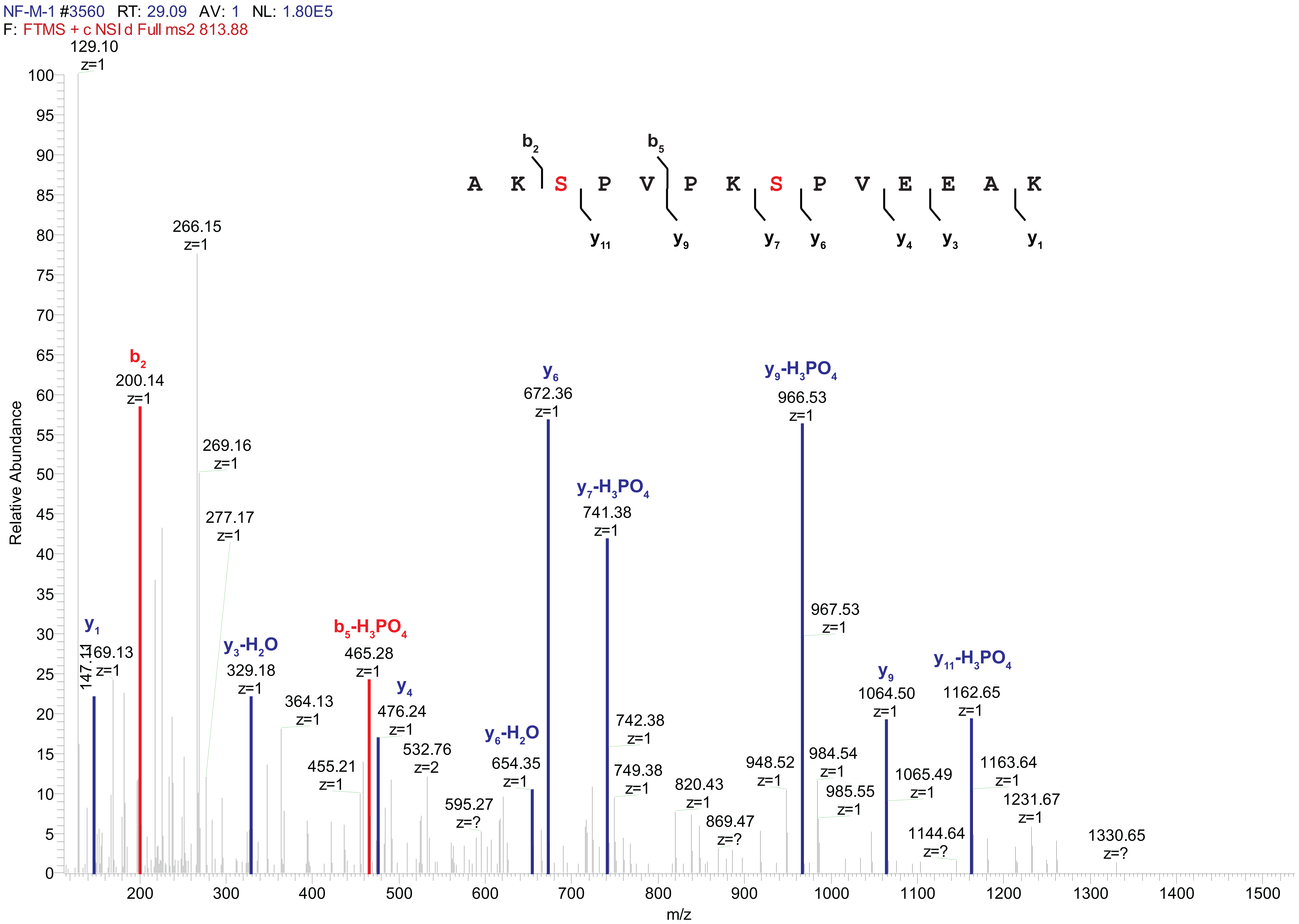
NF-M-1 #3560 RT: 29.09 AV: 1 NL: 1.80E5
F: FTMS + c NSI d Full ms2 813.88
A K S P V P K S P V E E A K
b2
b5
y11
y9
y7
y6
y4
y3
y1
Relative Abundance
m/z
129.10
z=1
266.15
z=1
b2
200.14
z=1
269.16
z=1
277.17
z=1
y1
147.11
169.13
z=1
y3-H2O
329.18
z=1
364.13
z=1
b5-H3PO4
465.28
z=1
455.21
z=1
y4
476.24
z=1
532.76
z=2
595.27
z=?
y6-H2O
654.35
z=1
y6
672.36
z=1
y7-H3PO4
741.38
z=1
742.38
z=1
749.38
z=1
820.43
z=1
869.47
z=?
948.52
z=1
y9-H3PO4
966.53
z=1
967.53
z=1
984.54
z=1
985.55
z=1
y9
1064.50
z=1
1065.49
z=1
1144.64
z=?
y11-H3PO4
1162.65
z=1
1163.64
z=1
1231.67
z=1
1330.65
z=?

Figure S1F

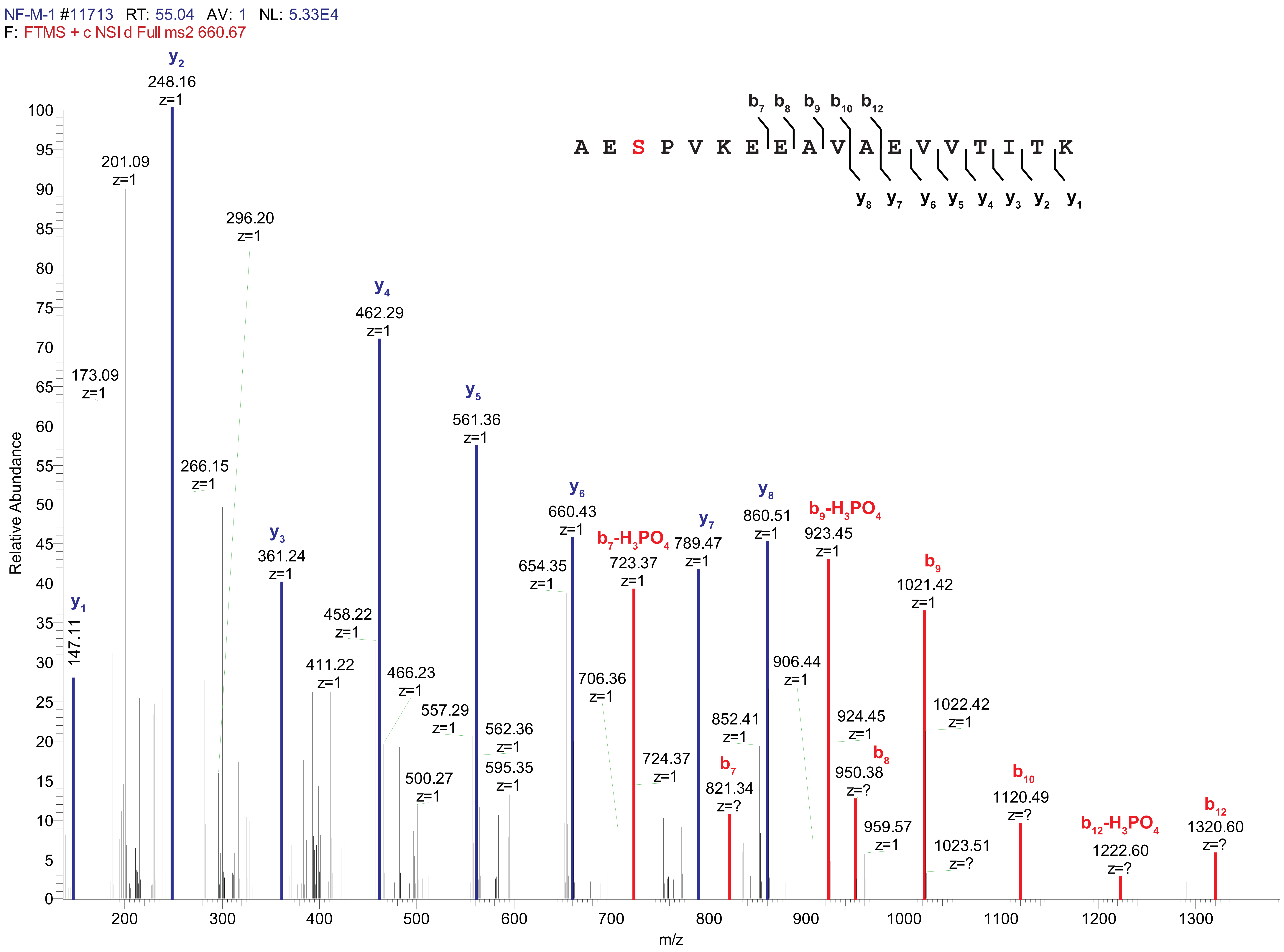
NF-M-1 #11713 RT: 55.04 AV: 1 NL: 5.33E4
F: FTMS + c NSI d Full ms2 660.67
A E S P V K E E A V A E V V T I T K
b7 b8 b9 b10 b12
y8 y7 y6 y5 y4 y3 y2 y1
Relative Abundance
m/z
y1 147.11
173.09 z=1
201.09 z=1
y2 248.16 z=1
266.15 z=1
296.20 z=1
y3 361.24 z=1
411.22 z=1
458.22 z=1
y4 462.29 z=1
466.23 z=1
500.27 z=1
557.29 z=1
y5 561.36 z=1
562.36 z=1
595.35 z=1
654.35 z=1
y6 660.43 z=1
706.36 z=1
b7-H3PO4 723.37 z=1
724.37 z=1
y7 789.47 z=1
b7 821.34 z=?
852.41 z=1
y8 860.51 z=1
906.44 z=1
b9-H3PO4 923.45 z=1
924.45 z=1
b8 950.38 z=?
959.57 z=1
b9 1021.42 z=1
1022.42 z=1
1023.51 z=?
b10 1120.49 z=?
b12-H3PO4 1222.60 z=?
b12 1320.60 z=?

Figure S1G

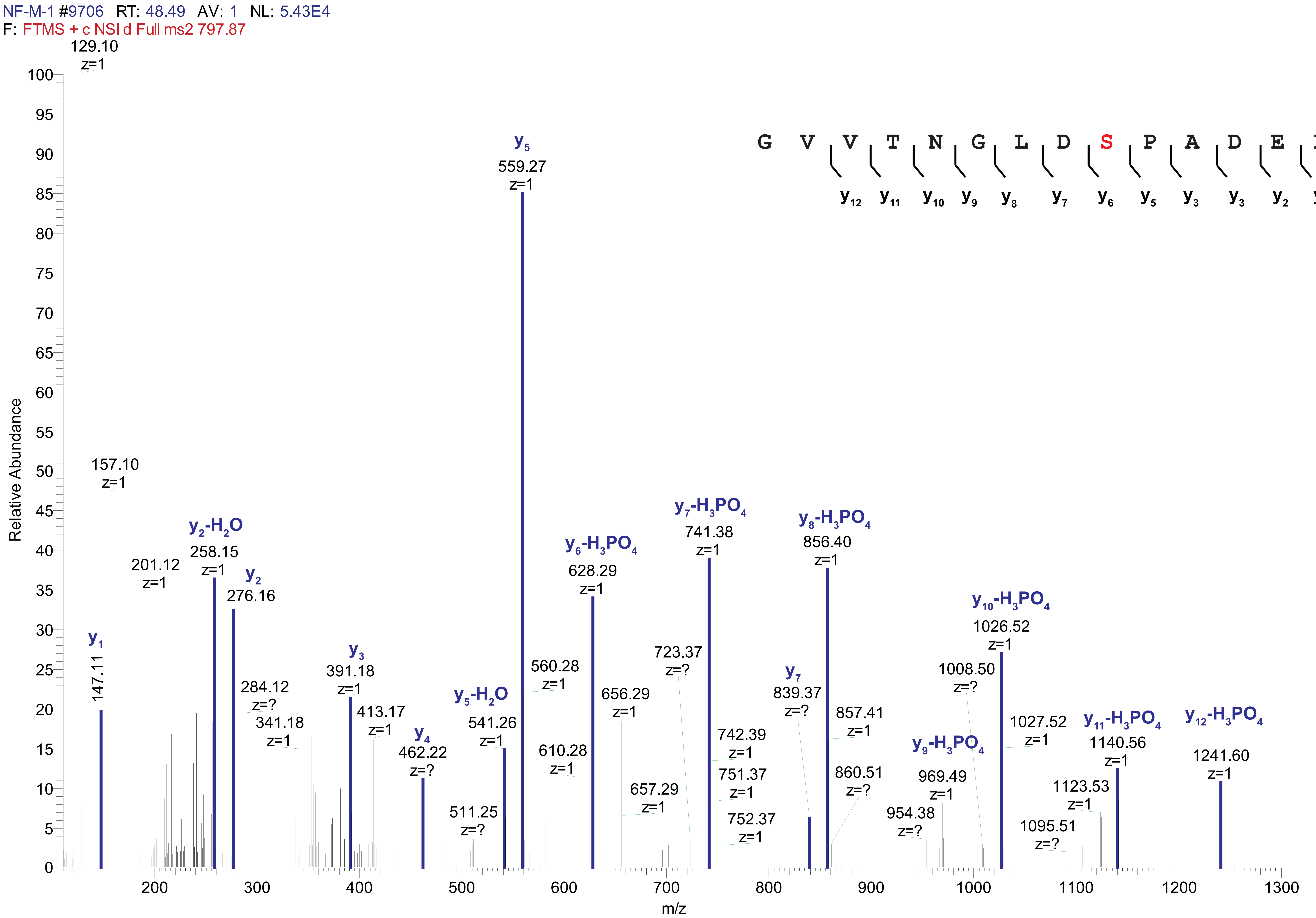
NF-M-1 #9706 RT: 48.49 AV: 1 NL: 5.43E4
F: FTMS + c NSI d Full ms2 797.87
G V V T N G L D S P A D E K
y12 y11 y10 y9 y8 y7 y6 y5 y3 y3 y2 y1
Relative Abundance
m/z
129.10 z=1
157.10 z=1
y1 147.11
201.12 z=1
y2-H2O 258.15 z=1
y2 276.16
284.12 z=?
341.18 z=1
y3 391.18 z=1
413.17 z=1
y4 462.22 z=?
511.25 z=?
y5-H2O 541.26 z=1
y5 559.27 z=1
560.28 z=1
610.28 z=1
y6-H3PO4 628.29 z=1
656.29 z=1
657.29 z=1
723.37 z=?
y7-H3PO4 741.38 z=1
742.39 z=1
751.37 z=1
752.37 z=1
y7 839.37 z=?
y8-H3PO4 856.40 z=1
857.41 z=1
860.51 z=?
954.38 z=?
y9-H3PO4 969.49 z=1
1008.50 z=?
y10-H3PO4 1026.52 z=1
1027.52 z=1
1095.51 z=?
1123.53 z=1
y11-H3PO4 1140.56 z=1
y12-H3PO4 1241.60 z=1

Figure S2

Ser336

GTKESLER**QLSDIEER**HNHDLSSYQDTIQQLENELRGTKWEMARHLREYQDLLNVKMALDIEI
AAYRKLLEGEETRFSTFAGSITGPLYTHRPPITISSKIQKPKVEAPKLKVQHKFVEEIIEETKVEDEK
SEMEEALTAITEELAVSMKEEKKEAAEEKEEEPEAEEEEVAAKKSPVKATAPEVKEEEGEKEEEE
GQEEEEEEDEGAKSDQAEEGGSEKEGSSEKEEGEQEEGETEAEAEGEEAEAKEEKKVEEKSEE
VATKEELVADAKVEKPEK **AKSPVPKSPVEEK** **GKSPVPKSPVEEK** **GKSPVPKSPVEEK**
GKSPVPKSPVEEK GKSPVSKSPVEEK **AKSPVPKSPVEEAK**
SKAEVGKGEQKEEEEKEVKEAPKEEKVEKKEEKPKDVPEKKK**AESPVKEEAVAEVVTITK**SVKV
HLEKETKEEGKPLQQEKEKEKAGGEGGSEEEGSDKGAKGSRKEDIAVNGEVEGKEEVEQETKE
KGSGREEEK**GVVTNGLDLSPADEK**KGGDKSEEKVVVTKTVEKITSEGGDGATKYITKSVTVTQ
KVEEHEETFEEKLVSTKKVEKVTSHAIVKEVTQSD Ser916

QLSDIEER – Ser346

AKSPVPKSPVEEK – Ser615/Ser620

GKSPVPKSPVEEK – Ser628/Ser633, Ser641/Ser646,Ser654/
Ser659 GKSPVSKSPVEEK **– Ser667/Ser672 – Not detected**

AKSPVPKSPVEEAK – Ser680/Ser685

AESPVKEEAVAEVVTITK – Ser736

GVVTNGLDLSPADEK – Ser837
